# Characterization of the gut microbiome and resistome of Galapagos marine iguanas (*Amblyrhynchus cristatus*) from uninhabited islands

**DOI:** 10.1186/s42523-022-00218-4

**Published:** 2022-12-14

**Authors:** Karla Vasco, Nataly Guevara, Juan Mosquera, Sonia Zapata, Lixin Zhang

**Affiliations:** 1 Department of Microbiology and Molecular Genetics, Michigan State University, East Lansing, MI 48824 USA; 2 Departamento de Procesos Químicos, Alimentos y Biotecnología, Universidad Técnica de Manabí, 130105 Portoviejo, Ecuador; 3 Galapagos Science Center, Universidad San Francisco de Quito, Quito, Ecuador; 4 Colegio de Ciencias Biológicas y Ambientales, Instituto de Microbiología, Universidad San Francisco de Quito, Quito, Ecuador; 5 Department of Epidemiology and Biostatistics, Michigan State University, East Lansing, MI 48824 USA

**Keywords:** Microbiome, Marine iguana, Galapagos, Resistome, Antibiotic resistance, Horizontal gene transfer, Metagenome

## Abstract

**Background:**

Understanding the natural microbiome and resistome of wildlife from remote places is necessary to monitor the human footprint on the environment including antimicrobial use (AU). Marine iguanas are endemic species from the Galapagos Islands where they are highly affected by anthropogenic factors that can alter their microbiota as well as their abundance and diversity of antimicrobial-resistant genes (ARGs). Thus, this study aims to apply culture-independent approaches to characterize the marine iguana’s gut metagenomic composition of samples collected from the uninhabited islands Rabida (*n* = 8) and Fernandina (Cabo Douglas, *n* = 30; Punta Espinoza, *n* = 30). Fresh feces from marine iguanas were analyzed through SmartChip RT-PCR, 16S *rRNA*, and metagenomic next-generation sequencing (mNGS) to identify their microbiome, microbial-metabolic pathways, resistome, mobilome, and virulome.

**Results:**

The marine iguana’s gut microbiome composition was highly conserved despite differences in ecological niches, where 86% of taxa were shared in the three locations. However, site-specific differences were mainly identified in resistome, mobilome, virulorome, and metabolic pathway composition, highlighting the existence of factors that induce microbial adaptations in each location. Functional gut microbiome analyses revealed its role in the biosynthesis and degradation of vitamins, cofactors, proteinogenic amino acids, carbohydrates, nucleosides and nucleotides, fatty acids, lipids, and other compounds necessary for the marine iguanas. The overall bacterial ARG abundance was relatively low (0.006%); nevertheless, the presence of genes encoding resistance to 22 drug classes was identified in the iguana’s gut metagenome. ARG-carrying contig and co-occurrence network analyses revealed that commensal bacteria are the main hosts of ARGs. Taxa of public health interest such as *Salmonella*, *Vibrio*, and *Klebsiella* also carried multidrug-resistance genes associated with MGEs which can influence the dissemination of ARGs through horizontal gene transfer.

**Conclusion:**

Marine iguanas depend on the gut microbiome for the biosynthesis and degradation of several compounds through a symbiotic relationship. Niche-specific adaptations were evidenced in the pool of microbial accessory genes (i.e., ARGs, MGEs, and virulence) and metabolic pathways, but not in the microbiome composition. Culture-independent approaches outlined the presence of a diverse resistome composition in the Galapagos marine iguanas from remote islands. The presence of AR pathogens in marine iguanas raises concerns about the dispersion of microbial-resistant threats in pristine areas, highlighting wildlife as sentinel species to identify the impact of AU.

**Supplementary Information:**

The online version contains supplementary material available at 10.1186/s42523-022-00218-4.

## Introduction

Marine iguanas (*Amblyrhynchus cristatus*) are endemic animals from the Galapagos Islands, Ecuador, specialized about 4.5 million years ago to be the world’s only sea lizards able to survive by grazing red and green algae [1]. With a population of approximately 19,800–210,000 specimens, these reptiles are vulnerable to extinction due to anthropogenic factors including climatic change, non-native predators, and plastic and oil pollution [2]. Therefore, multidisciplinary efforts are needed to preserve this iconic species. Climate warming could greatly affect iguana populations due to ecological perturbations which could result in a lack of food availability, diseases, and metabolic adaptations. Such is the case of El Niño events that dropped 70% of some populations in 1982–83 [3], and that was associated with changes in bone metabolism of the marine iguanas to enable these animals to shrink to survive harsh conditions [4, 5]. Likewise, environmental changes can alter the microbiome composition and affect its role in the iguana’s health. Though, little research has been conducted to characterize the microbiota composition and microbial-associated metabolic pathways in the marine iguana’s gut. Prior studies compared the microbiome of marine and land iguanas from the Galapagos Islands to explore differences in ecological niches [6–8]. However, these prior studies have failed to evaluate a representative number of samples and were conducted about a decade ago when the availability of databases and bioinformatic pipelines was limited. New sequencing technologies, bioinformatics approaches, and more comprehensive databases would enable a better characterization of the microbiome composition of this endangered species.

Furthermore, the proximity between endemic animals and human populations in the Galapagos could affect the number and diversity of antimicrobial-resistant genes (ARGs), a collection of genes known as resistome. The Galapagos National Park reported about 275,000 visitors from around the globe each year; meanwhile, the resident population corresponds to 25,244 inhabitants [9] that occupy 3% of the landmass in the archipelago which could impact native and endemic fauna. Antibiotic usage (AU) in humans and domestic animals has been associated with the emergence of multidrug-resistant bacteria [10] which are considered one of the top ten public health threats, causing about 4.95 million human deaths and billions of dollars in healthcare costs worldwide [11]. Wildlife from uninhabited islands, such as marine iguanas, could provide a model for remote settings where animals have no history of antibiotic exposure or contact with introduced species necessary to evidence the impacts of AU in pristine areas. A previous study evidenced that dominant Enterobacteria from Galapagos land iguanas (*Conolophus pallidus*) living on an uninhabited island lacked resistance to nine antimicrobials including ampicillin, tetracycline, chloramphenicol, streptomycin, kanamycin, gentamicin, amikacin, nalidixic acid, and trimethoprim/sulfamethoxazole [12]. Similarly, no resistance was found in *Escherichia coli* and *Salmonella enterica* isolated from marine iguanas [13, 14]. Though tetracycline resistance genes have been detected in total fecal DNA from marine iguanas (*n* = 5) including *tetM*, *tetO*, *tetS*, and *tetW* [15]. Similarly, metagenomic analyses of 8 marine iguanas from San Cristobal and Lobos islands found that the most abundant ARG classes were bacitracin, beta-lactams, and macrolide-lincosamide-streptogramin (MLS) [16]. Classifying microbial reservoirs of ARGs in the marine iguana’s gut is also key to comprehending how these genes disseminate in bacterial populations and their potential threat to animal and human health, something that needs to be done.

Culture-independent approaches such as 16S *rRNA* sequencing, RT-PCR, and metagenomic-next-generation sequencing (mNGS) of Galapagos marine iguana feces are essential to characterize their microbiome and resistome composition. In this study, we aimed to analyze the fecal metagenome of three colonies of marine iguanas located in two uninhabited islands, Rabida and Fernandina, each one with different subspecies of marine iguanas: *A. cristatus wikelskii* and *A. cristatus cristatus*, respectively [17]. These unique and rarely studied sites were included since access was granted to a collection of fecal samples obtained during a research cruise carried out in 2018. Rabida is a 5 km^2^ island located in the middle of the archipelago where different oceanic currents converge including Cromwell, Panama, South Equatorial, and Peruvian. Fernandina is the youngest and most pristine island of the archipelago, where two spots were chosen: Punta Espinoza which is the only visitor place in Fernandina located on the eastern side of the island, and Cabo Douglas located on the western side. Differently from Rabida, Fernandina is only surrounded by the Cromwell oceanic current since Isabela Island acts as a barrier for the other currents. The Cromwell current brings cold nutrient-rich waters that supports a highly diverse marine ecosystem [18]. Here we analyzed the microbiome composition and its associated metabolic pathways, as well as the resistome and the interactions between mobile genetic elements (MGEs), virulence gene, ARGs, and bacterial taxa. Surveillance of the wildlife core microbiome and resistome in remote settings will help to identify microbial communities associated with healthy animals and evidence anthropogenic impacts over time.

## Materials and methods

### Sampling collection and DNA extraction

**Fig. 1:**
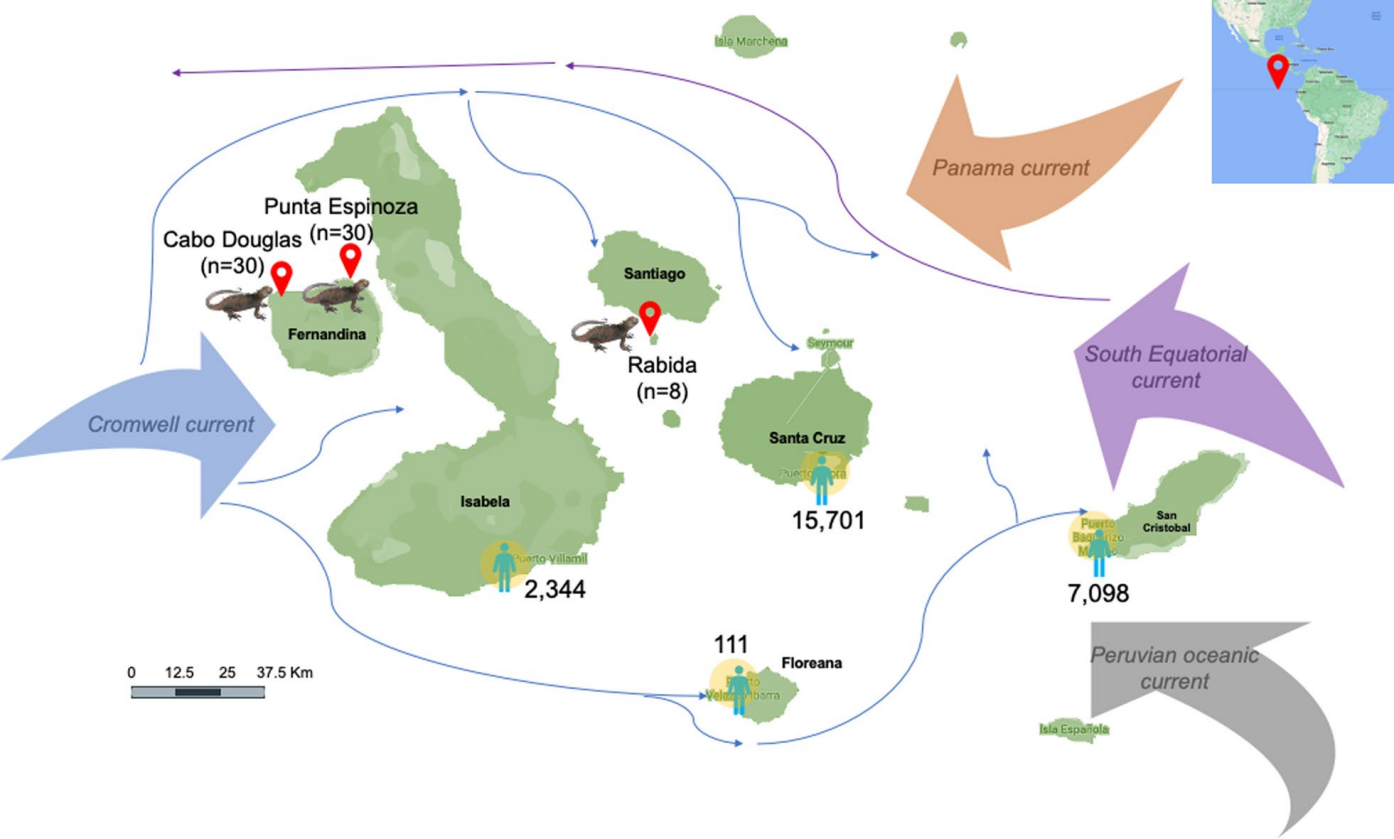
Geographic location of the sampling sites in the Galapagos Islands. The main current systems and the human population of the archipelago are shown

Samples were collected on the central island of Rabida (latitude 0.401781, longitude 90.712194), and the western island of Fernandina at Punta Espinoza (latitude 0.263846, longitude 91.445559) and Cabo Douglas (latitude 0.303372, longitude 91.652145) from July 18 to 20, 2018 during the cold-dry season (Fig. 1). Fecal droppings were taken from 68 marine iguanas in the three sites: Punta Espinoza (*n* = 30), Cabo Douglas (*n* = 30), and Rabida (*n* = 8). Samples from these locations were collected during a research cruise carried out in 2018 where a convenience sampling was carried out. The fecal samples were preserved in 96% ethanol, transported in dry ice, and stored at − 80 °C until DNA extraction. Fecal DNA was extracted with the DNeasy PowerSoil Pro Kit (Qiagen®) and analyzed with a Qubit Fluorometer (Invitrogen™) and NanoDrop 1000 Spectrophotometer (Thermo Fisher Scientific) to test the quality and quantity of the genomic DNA.

### Amplicon library processing

The V4 hypervariable region of the 16S *rRNA* gene from each DNA sample (*n* = 68) was amplified using Illumina compatible, dual indexed primers 515f/806r following the protocol developed in the Schloss lab [19]. PCR products were batch normalized using Norgen Biotek NGS Normalization Kit and products recovered were pooled and cleaned using AmpureXP magnetic beads. The pooled amplicons were sequenced on Illumina MiSeq in a 2 × 250 bp paired-end format. The amplicon preparation and next-generation sequencing were carried out at the Michigan State University Genomics Core. Quantitative Insights Into Microbial Ecology 2 (QIIME2) [20] pipeline was used for demultiplexing with Casava 1.8 paired-end and denoising with DADA2 [21]. Operational taxonomic units (OTUs) were clustered de novo with 97% similarity using VSEARCH [22] and the taxonomy was assigned with the SILVA database (v138) [23]. Finally, a phylogenetic tree of the 16S *rRNA* sequences was constructed with SATé-enabled phylogenetic placement (SEPP) trees [24].

### Microbiome analyses of 16S *rRNA* sequences

The R packages Phyloseq v.1.24.2 [25] and Vegan v.2.5.6 [26] were used to estimate the OTU richness and composition with the indices Shannon and Chao1 for alpha-diversity and the beta-diversity was calculated with Bray-Curtis dissimilarity and Weighted Unifrac distances [27]. Wilcoxon and Kruskal-Wallis tests were used to compare alpha diversity indices among locations. While differences in the microbial profiles were analyzed with permutational multivariate analysis of variance (PERMANOVA) with 999 permutations and with permutational analysis of multivariate dispersion (PERMDISP). Differentially abundant taxa among locations were analyzed with Analysis of compositions of microbiomes with bias correction (ANCOM-BC) [28] and Microbiome Multivariable Associations with Linear Models (MaAsLin2) [29].

### Resistome and mobile detection with SmartChip RT-PCR

The quantification of 53 MGEs and 321 ARGs (Additional file 1) was carried out through SmartChip RT-PCR (Takara BIO/Wafergene, USA) using previously validated primers and protocol [30–32]. The estimated genomic copies (GC) of ARGs and MGEs were computed with the formula (1) considering a cycle threshold (Ct) cutoff of 32. The relative abundance (RA) was calculated by dividing the GC of a given gene by the GC of the 16S *rRNA* gene, as shown in formula (2). Shannon index, Bray Curtis dissimilarity, and differentially abundant features were calculated as described for the microbiome analyses.

1 $$GC = 10^{{\left[ {\left( {32 - Ct} \right)/3.333} \right]}}$$

2 $$RA = \frac{{GC_{ARG} }}{{GC_{16S rRNA} }}$$

### Metagenomic next-generation sequencing (mNGS)

A subset of 32 samples (Punta Espinoza, *n* = 12 (random); Cabo Douglas, *n* = 12 (random); Rabida, *n* = 8) was selected for metagenomic next-generation sequencing (2 × 150 bp) in a HISEQ 4000 to an average depth of 2.46 GB per sample. First, raw sequences were analyzed with FastQC [33]. Adapters and bad-quality sequences were removed with Trimmomatic [34]. Taxonomic classification of the trimmed metagenomic reads was carried out with Kraken2 [35] and the relative abundance was computed with Bayesian Reestimation of Abundance with KraKEN (Bracken) [36]. Krona [37] was used to visualize the metagenomic composition using the average fraction calculated with Bracken. While Kraken Tools were used to calculate the Shannon index and the Bray-Curtis dissimilarly matrix of the bracken results, which were further analyzed as mentioned for the microbiome analyses. MetaSPAdes [38] was applied to assemble contigs from paired trimmed sequences and the assemblies were translated to amino acid sequences with the PROkaryotic DYnamic programming Gene-finding ALgorithm (Prodigal) for further analyses [39].

### Functional microbial profile

Paired and trimmed short reads were merged with PEAR (Paired-End reAd mergeR) 0.9.8 [40] and the resulting assembled reads were used for functional profiling with BioBakery3 [41]. This integrated pipeline includes methods to identify MetaCyc metabolic pathways [42] and their taxonomic profiling through HUMAnN 3.0 and MetaPhlAn3 using the databases ChocoPhlAn and Uniref90 [43]. Differentially abundant pathways between locations were identified with ANCOM-BC [28].

### Resistome, mobilome, and virulome of mNGS

Assembled contigs were analyzed with DeepARG [44] to identify ARGs and potential ARGs, a machine learning approach that predicts with better recall and precision ARGs. MGEs and genes involved in horizontal gene transfer (HGT) were identified with the protein database mobileOG [45], while virulence genes were identified with the protein database VFDB [46]. The open-reading frames (ORFs) of translated sequences were mapped to the protein databases using Diamond v.2.0.1. setting the best hit for the identification of genes [47]. The abundance of ARGs, MGEs, and virulence genes was normalized by calculating the proportion of gene reads based on the number of bacterial reads of the assembled contigs identified with Kraken2. The normalized counts were used to calculate the alpha diversity with the indices Shannon and Chao1 and the beta diversity with Bray-Curtis dissimilarity as described for the microbiome analysis.

### ARG carrying contig analyses

ARG-carrying contigs (ACCs) identified with DeepARG were extracted with *seqtk*. Then, the taxonomy of the sequences was analyzed with the contig analyzer tool (CAT) [48] which uses a voting approach of ORFs identified in the contigs at the entire taxonomic lineage. The presence of MGEs and virulence genes in the ACCs were extracted from the mobilome and virulome results previously described by selecting contigs by their identification number (qseqid) for each sample. The frequency of taxa at the phylum and genus levels, as well as the number of contigs carrying MGEs and virulence genes, was carried out with all ACCs. A co-occurrence network based on correlations equal to or higher than 0.5 and a p-value lower than 0.01 calculated with a matrix of presence/absence of ARGs, MGEs, and virulence genes identified in ACCs was built in Gephi to evidence common contig associations.

### Network analysis of 16S *rRNA* sequences and SmartChip amplicons

The normalized abundances of ARGs and MGEs identified with SmartChip and bacterial genera classified with 16S *rRNA* sequencing were used to find associations within the marine iguana’s gut microbiome. Spearman correlations were calculated between the relative abundance of bacterial genera and the normalized GC of ARGs and MGEs in R 4.0.5 with the package Hmisc [49]. Correlations equal to or higher than 0.75 and a p-value lower than 0.01 were used to construct Fruchterman-Reingold networks with Gephi v.0.9.2 [50].

### Global metagenomic network

A matrix consisting of the normalized abundance of ARGs, MGEs, virulence genes, and bacterial genera of the 32 marine iguana samples analyzed through mNGS was used to calculate Spearman correlations in R 4.0.5 with the package Hmisc [49]. Correlation coefficients equal to or higher than 0.75 with a p-value lower than 0.01 were filtered and used to build Fruchterman-Reingold networks with Gephi v.0.9.2 [50]. Network statistics were calculated with Gephi including degree of centrality, modularity, clustering coefficient, and number of connected components. Ego networks of bacterial genera of public health interest were extracted from the global network, including *Salmonella*, *Vibrio*, *Shigella*, *Enterobacter*, *Escherichia*, *Enterococcus*, *Streptococcus*, *Helicobacter*, *Bordetella*, among others.

### Core metagenome

The presence of different taxa, ARGs, MGEs, virulence genes, and metabolic pathways was compared among the three marine iguana colonies to identify modifications related to different ecological niches. The presence of features (taxa or gene) in at least one sample per location was identified and compared among sites using Venn diagrams with the R package VennDiagram v.1.7.1 [51].

## Results

### Microbiome diversity

**Fig. 2:**
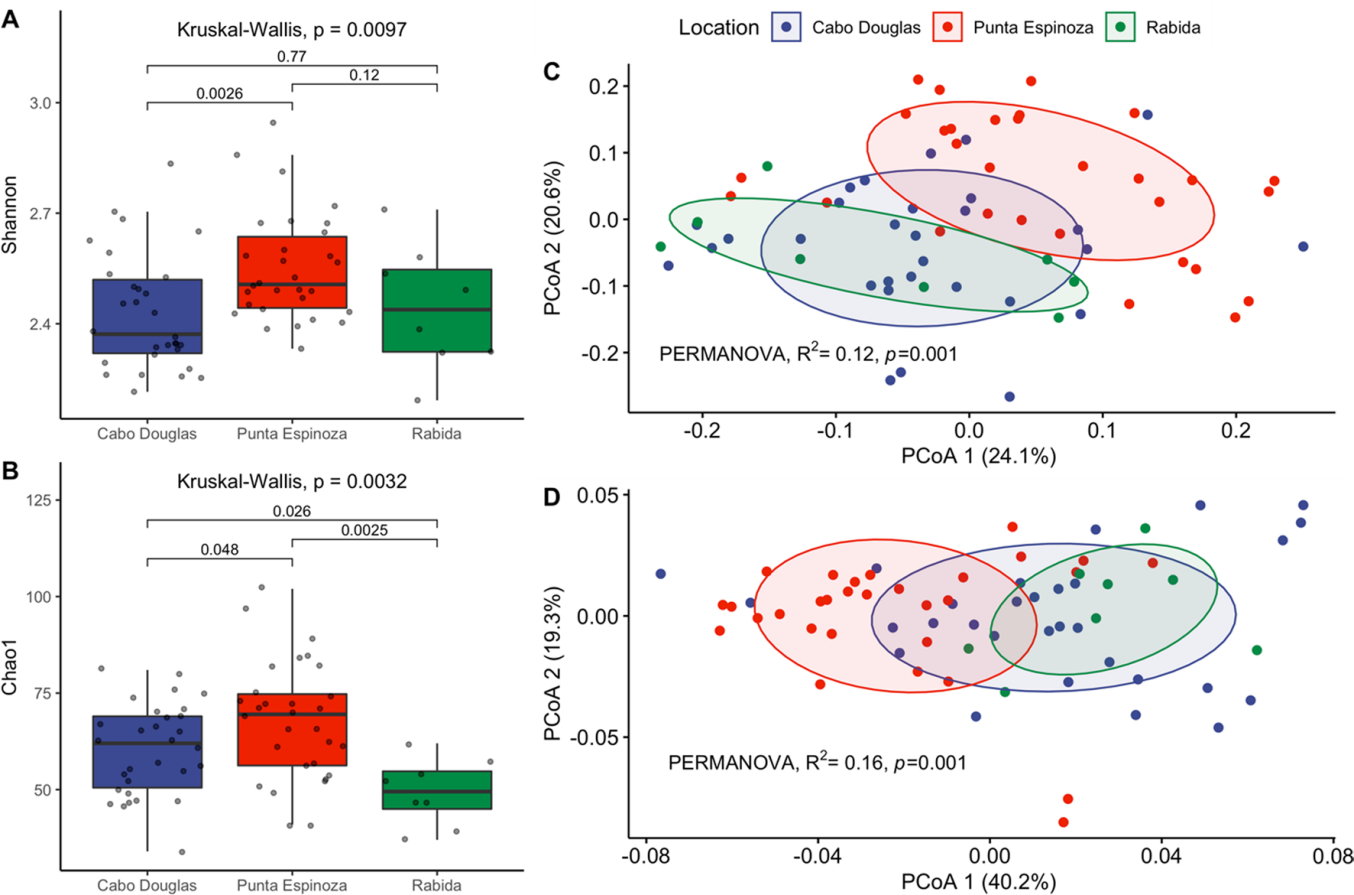
Microbiome alpha and beta diversity from 16 s *rRNA* sequences. Alpha diversity was measured with Shannon (**A**) and Chao1 (**B**) indexes. Wilcoxon test was used to compare the values between locations. Beta diversity was calculated with Bray-Curtis dissimilarity matrix (**C**) and Weighted Unifrac (**D**), which were examined with Principal Coordinate Analysis (PCoA). The ellipses in C and D contain at least 70% of the samples per location

16S *rRNA* sequencing and mNGS identified that gut microbiome of marine iguanas from Rabida and Cabo Douglas had similar microbiome diversity while iguanas from Punta Espinoza had the highest alpha diversity and a different mean composition among the three sites (Fig. 2; Additional file 2: Fig. S1). Rabida had a lower OTU richness compared to samples from Fernandina (*Chao1*, *P* < 0.03) (Fig. 2B). Interestingly, Bray-Curtis and Weighted Unifrac PCoAs show that marine iguanas from Cabo Douglas and Rabida shared a similar gut microbiome composition even though these are the most geographically distant sites. The microbiome of marine iguanas from the three locations had similar variability among individuals (PERMDISP, *P* = 0.27), but Punta Espinoza had a different mean composition (PERMANOVA, *P* = 0.001) (Fig. 2C, D).

### Fecal microbiome composition

On average, the gut microbiome of the Galapagos marine iguanas was composed mainly by dominion bacteria which comprised 99.11% of the reads, while the remainder were eukaryote (0.5%), archaea (0.3%), and viruses (0.09%). The fungi Basidiomycota were the only eukaryotic organisms identified (0.5%). The most abundant archaea were Methanobacteria (0.08%), Methanococci (0.06%), Thermoprotei (0.05%) and Methanomicrobia (0.05%). While most of the viruses were Caudovirales (0.06%), Bamfordvirae (0.02%), and Bunyavirales (0.003%).

**Fig. 3:**
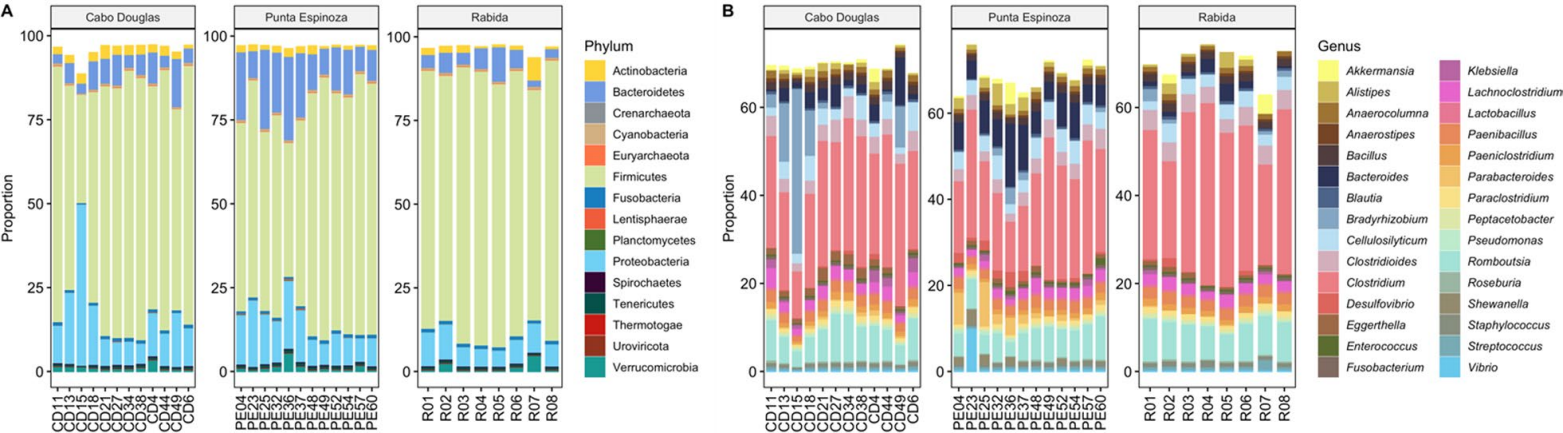
Gut microbiome composition (shot-gun sequencing) of marine iguanas from Fernandina and Rabida Islands. **A** Bar plot showing the proportion of the top 15 phyla by location. **B** Bar plot showing the proportion of the top 30 most abundant genera by location

Firmicutes was the most abundant bacterial phylum (69.4%), followed by Proteobacteria (11.9%), Bacteroidetes (9.08%), and Actinobacteria (2.18%) (Fig. 3A). Through 16S *rRNA* sequencing, however, the microbiome was dominated by the phylum Firmicutes (97.3%) showing important differences based on the sequencing method (Additional file 2: Fig. S2). The five most abundant bacterial genera identified through mNGS were *Clostridium* (25.8%), *Romboustia* (7.35%), *Bacteroides* (4.09%), *Clostridioides* (3.64%) and *Cellulosilyticum* (3.061%) (Fig. 3B).

Dissimilarities in the mean metagenomic composition were explained by the differential abundance of several taxa among locations, shown in detail in Additional file 3. Out of 9280 taxa identified in the marine iguana’s metagenome, a total of 157 were differentially abundant between Punta Espinoza and Rabida, 89 between Cabo Douglas and Punta Espinoza, and 17 between Cabo Douglas and Rabida (Additional file 3).

### Functional microbiome profile

**Fig. 4:**
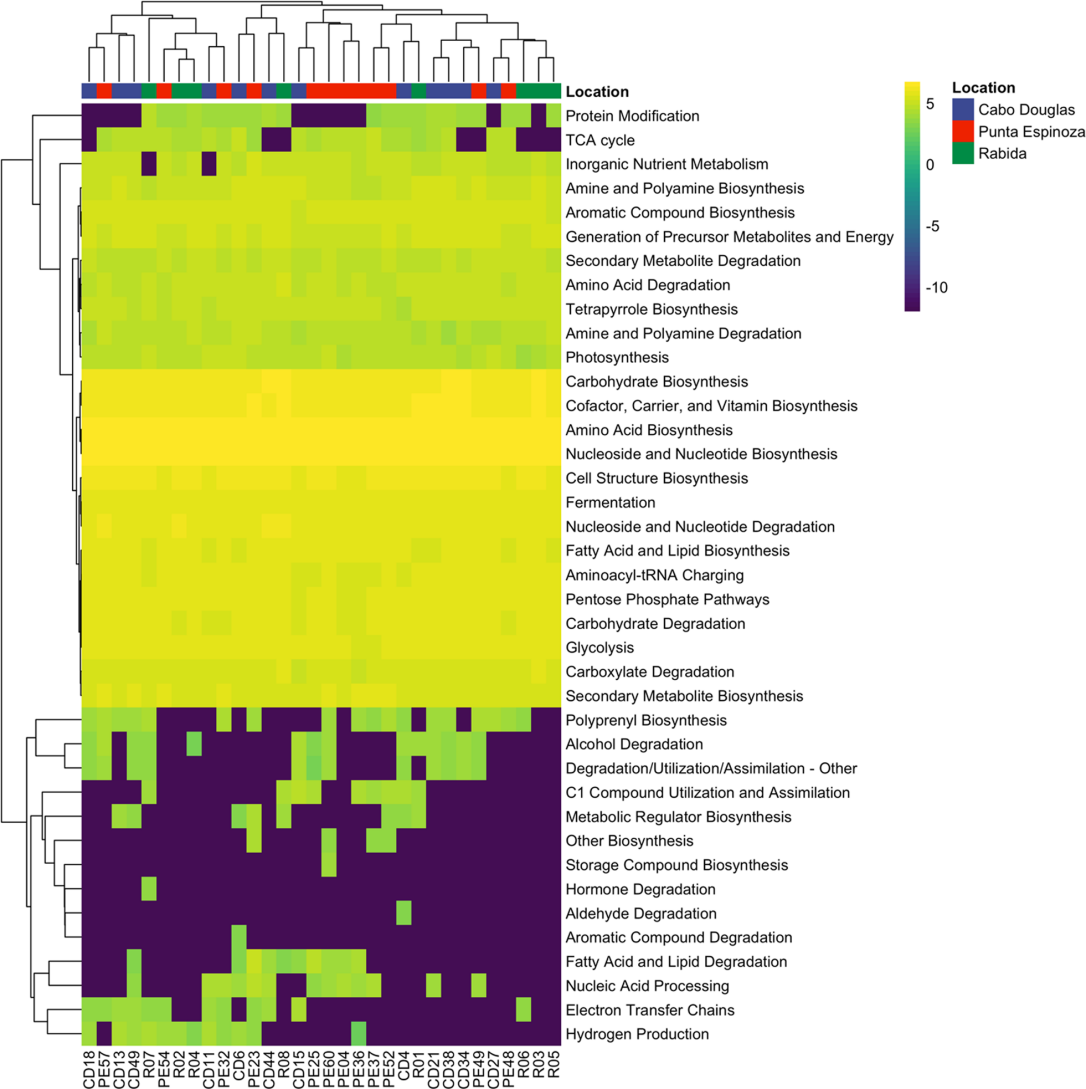
Heatmap of the functional gut microbiome profile of the Galapagos marine iguanas. The abundance of each pathway subclass is shown as the logarithm 10 of the proportion of reads. Hierarchical clustering with the method Ward was used to aggregate samples and metabolic pathways

A total of 353 MetaCyc metabolic pathways were identified through the Biobakery3 pipeline (Additional file 4). Most of the microbial pathways allow biosynthesis (*n *= 205) and degradation/utilization/assimilation (*n* = 100) of different compounds. Furthermore, other pathways enable the generation of precursor metabolites and energy (*n* = 45) and macromolecule modification (*n* = 3). At the subclass level, the top 10 most abundant pathways were nucleoside and nucleotide biosynthesis (24.04%), amino acid biosynthesis (18.55%), cofactor, carrier, and vitamin biosynthesis (10.82%), carbohydrate biosynthesis (10.46%), cell structure biosynthesis (5.91%), nucleoside and nucleotide degradation (4.15%), fermentation (3.57%), glycolysis (2.95%), carbohydrate degradation (2.81%), pentose phosphate pathways (2.62%), fatty acid and lipid biosynthesis (2.53%), and aminoacyl-tRNA charging (2.39%) (Fig. 4).

Punta Espinoza had the highest number of metabolic pathways as compared to the other two locations (Additional file 2: Fig. S3). Similarly, while Cabo Douglas and Rabida shared the same mean pathway composition, Punta Espinoza had a significantly different average functional microbial profile (PERMANOVA, R^2^ 0.2, *P* = 0.005) (Additional file 2: Fig. S3). Nevertheless, at the subclass level all locations shared similar functional microbiome, evidenced by hierarchical clustering showing a random aggrupation of samples from different locations (Fig. 4).

Among the biosynthesis subclass, multiple pathways resulted in cofactor, carrier, and vitamin biosynthesis (*n* = 55) which included flavin, folate, thiamine, vitamins B6, B7, B12, C, heme, NAD, coenzyme A, electron carriers, single carbon carriers, and molybdenum-containing cofactors. The amino acid biosynthesis pathways (*n* = 43) predominantly included proteogenic amino acids (*n* = 29). Other pathways included the biosynthesis of nucleoside and nucleotides (*n* = 32), carbohydrates (*n* = 20), fatty acids and lipids (*n* = 17), cell structures (*n* = 9), secondary metabolites (*n* = 9), amine and polyamines (*n* = 8), tetrapyrroles (*n* = 5), aromatic compounds (*n* = 2), aminoacyl-tRNA charging (*n* = 1), metabolic regulator (*n* = 1), polyprenyl (*n* = 1), storage compound (*n* = 1), and other (*n* = 1).

The gut microbiome of marine iguanas is capable to degrade several compounds such as carbohydrates, including polysaccharides (pathways, *n* = 7) and sugars (*n* = 14), carboxylates (*n* = 17), nucleoside and nucleotides (*n* = 13), fatty acids and lipids (*n* = 7), aromatic compounds (*n* = 7), amine and polyamines (*n* = 5), proteinogenic amino acids (*n* = 7), reduction of nitrogen (*n* = 4) and sulfur compounds (*n* = 3), among others molecules. Furthermore, gut microorganisms carry pathways to ferment (*n* = 17) pyruvate, and alcohols, and to produce short-chain fatty acids including butanoate, lactate, acetate, and propanoate.

Additionally, the metabolic pathways were assigned to 25 species and unclassified bacteria (Additional file 2: Fig. S4). *Vibrio alginolyticus* was the species with the highest number of metabolic pathways (*n* = 124), followed by *Shewanella algae* (*n* = 71), *Parabacteroides distasonis* (*n* = 66), *Bacteroides ovatus* (*n* = 60), *Klebsiella pneumoniae* (*n* = 55), *Eggerthella lenta* (*n* = 45), *Vibrio antiquaries* (*n* = 38), *Alistipes indistinctus* (*n* = 32), *Photobacterium damselae* (*n* = 22), *Salmonella enterica* (*n* = 21), *Alistipes shahii* (*n* = 21), *Butyricimonas synergistica* (*n* = 19), among others (Additional file 2: Fig. S4).

**Fig. 5:**
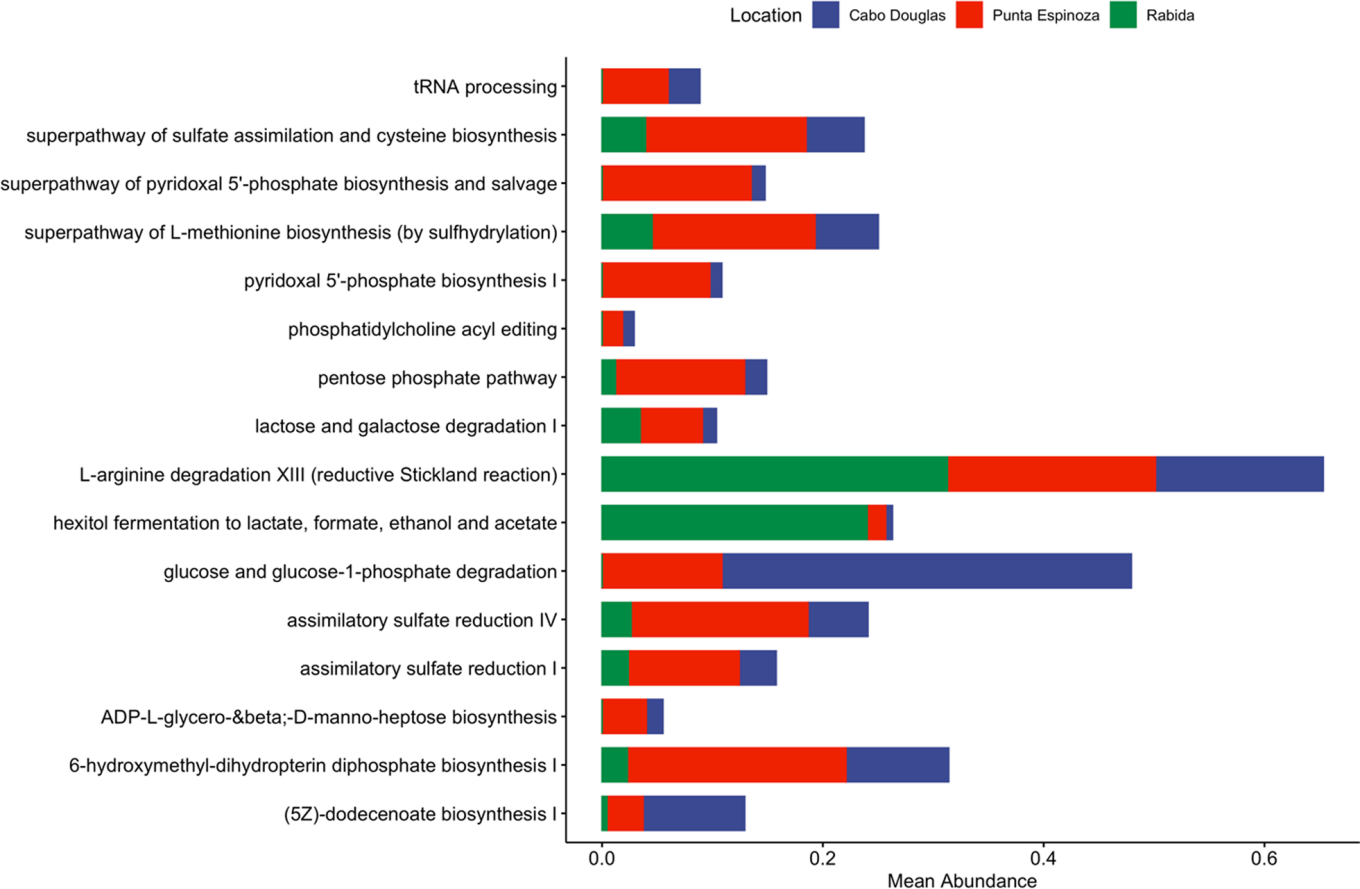
Differentially abundant metabolic pathways of the marine iguana’s gut from three different locations. Abundance is shown as the average relative abundance

Significant differences among locations were observed in 16 pathways with ANCOM-BC (Fig. 5). Notably, samples from Rabida had a higher abundance of ‘hexitol fermentation to lactate, formate, ethanol and acetate’ as compared to samples from Fernandina. Likewise, iguanas from Fernandina Island had a higher abundance of ‘glucose and glucose-1-phosphate degradation’, ‘pyridoxal 5’-phosphate biosynthesis I’, ‘superpathway of pyridoxal 5’-phosphate biosynthesis and salvage’, ‘tRNA processing’, ‘ADP-L-*glycero*-β-D-*manno*-heptose biosynthesis’, and ‘phosphatidylcholine acyl editing’ than iguanas from Rabida (Fig. 5).

### Resistome, mobilome, and virulome composition

A total of 125 ARGs and 23 MGEs were identified with SmartChip RT-PCR in the 68 fecal samples of marine iguanas. On average, 0.02% and 0.0006% of the normalized genomic counts corresponded to the targeted ARGs and MGEs, respectively. The most abundant genes were aminoglycoside ARGs, MGEs, and multidrug-resistant genes (MDR) (Additional file 1). Clinically relevant ARGs were also identified in marine iguana samples including genes encoding for extended-spectrum-beta-lactamases (ESBL), colistin, vancomycin, fluoroquinolone, and sulfonamide resistance (Additional file 1).

Metagenomic analyses of assembled contigs from 32 fecal samples revealed the presence of additional 185 ARGs and 1034 genes implicated in HGT not identified through RT-PCR. Additionally, 670 microbial virulence genes were identified with the VFDB database (Additional file 5). The normalized abundance of these genes showed an average proportion of 0.03% of genes involved in horizontal gene transfer (HGT), 0.006% of ARGs, and 0.007% of virulence genes. Differences in the proportion of MGEs and ARGs between RT-PCR and mNGS reflect biases based on methodology and normalization; however, both technologies were applied since mNGS is considered more comprehensive for the identification of these genes, while RT-PCR is more sensitive to detect clinically important ARGs and MGEs.

**Fig. 6:**
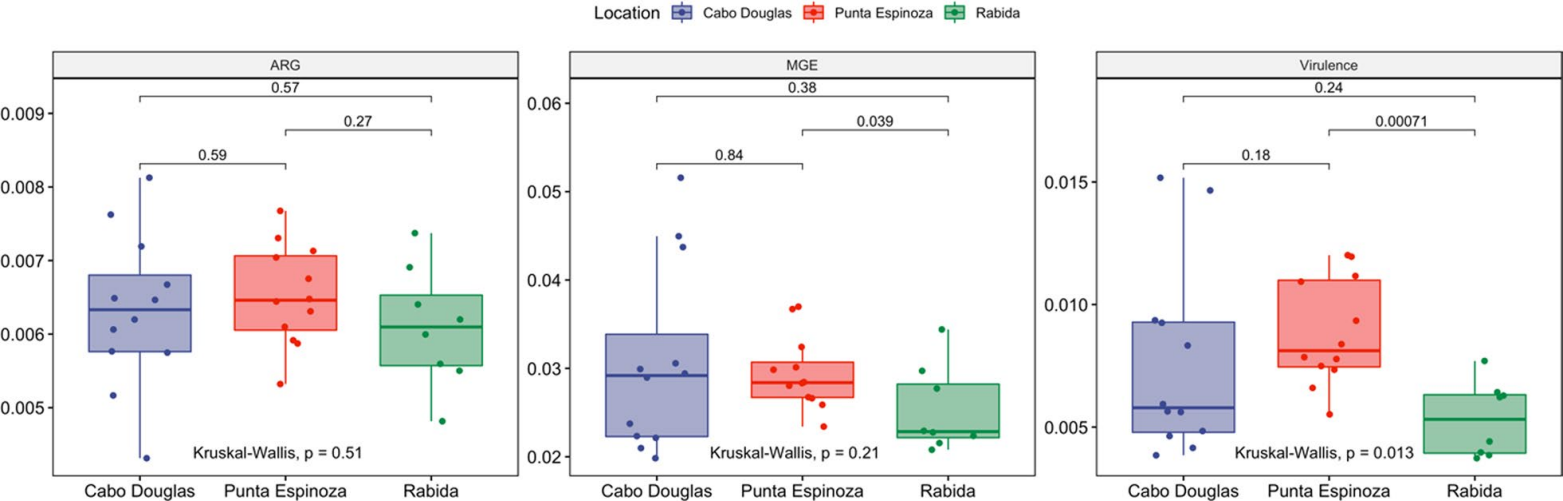
Resistome, mobilome and virulome normalized proportion in the marine iguana’s gut metagenomes

Significant differences were observed in the proportion of ARGs, MGEs and virulence genes between sites, with iguanas from Punta Espinoza carrying a higher abundance of MGEs and virulence genes as compared to Rabida (*P* < 0.04) but not in the number of ARGs (*P* > 0.2) (Fig. 6). Diversity metrics showed that Cabo Douglas and Rabida have similar richness and composition of resistome (*P* = 0.7), mobilome (*P* = 0.07), and virulome (*P* > 0.14) (Additional file 2: Figs. S5–S7). While Punta Espinoza had a higher richness and different mobilome, resistome, and virulome composition as compared to the other two sites (*P* < 0.05) (Additional file 2, Figures S5-S7). Likewise, the Shannon index of ARGs and MGEs identified with RT-PCR was similar between the three locations (*P* = 0.73) (Additional file 2: Fig. S8A); nevertheless, the number of observed genes was higher in samples from Cabo Douglas as compared to Rabida (*P* = 0.034) (Additional file 2: Fig. S8B). The resistome/mobilome composition analyzed with Bray–Curtis dissimilarity showed overlapping clustering among sampling sites with some differences in the mean composition (Additional file 2: Fig. S8C) (PERMDISP, *F* = 2.05, *P* = 0.14; PERMANOVA, *R*^*2*^ = 0.08, *P* = 0.001).

**Fig. 7:**
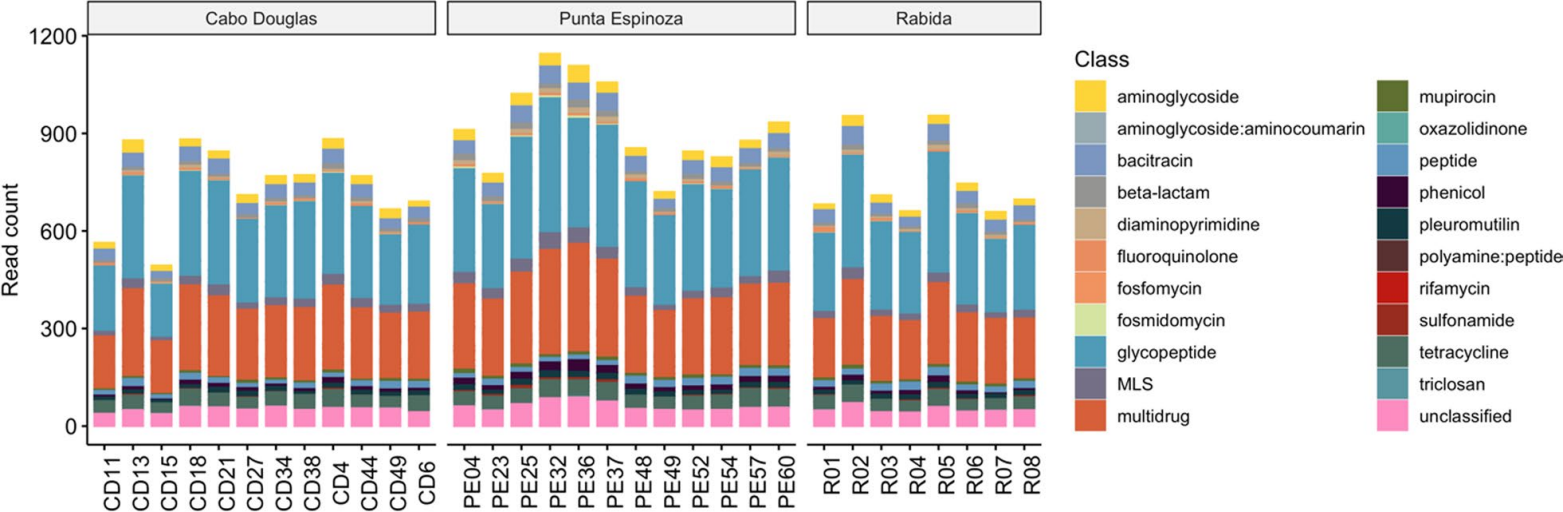
Gut resistome composition of the Galapagos marine iguanas. The abundance is shown as number of reads in assembled contigs

The resistome identified through mNGS was composed of 22 classes of ARGs, dominated by glycopeptide (36.3%) and MDR (28.8%) (Fig. 7). Other representative ARG classes were bacitracin (5.83%), tetracycline (5.43%), MLS (3.38%), aminoglycoside (2.9%), and phenicol (2.42%) (Fig. 7). Among genes implicated in HGT, fifteen classes were identified, predominantly genes involved in replication/recombination/repair (99.8%), followed by phages (0.07%), stability/transfer/defense (0.04%), and plasmid genes (0.03%). Finally, about 33.3% of the virulence genes identified were associated with immune modulation, including genes encoding for capsule (22.04%) and LPS (6.5%) as the most abundant. Adherence genes (21.78%), nutritional/metabolic factors (10.73%), and stress survival (7.51%), among other virulence mechanisms were also identified (Additional file 5).

Nearly sixty-four percent of normalized ARG-genomic counts identified with SmartChip belong to the aminoglycoside ARG *acc3-*Via, followed by *czcA* (MDR; 13%), *vanC2/C3* (vancomycin; 5.7%), *tetPA* (tetracycline; 2.9%), and other ARGs found in lower proportions (Additional file 2: Fig. S9). The most abundant MGEs were *intI1F165* (integron; 77.5%), *trb-C* (plasmid; 8.8%), *IncP-oriT* (plasmid; 5%), and *IS200* (insertion sequence; 0.4%). Among sites, Punta Espinoza had the highest abundance of ARGs from the classes fluoroquinolone (*qnrA*), *tolC* (MDR), *IncP-oriT*, and sulfonamide ARGs as well as the lowest abundance of *tetPA*, *trbC*, and *Intl3*. Rabida had the highest abundance of *tetpA*, *aadA7*, *qepA1-2*, *czcA* and the lowest abundance of the aminoglycoside genes *aac3* and *aph6ia*. Finally, Cabo Douglas had the lowest abundance of the *aac(6’)-is-iu-ix* and the highest abundance of *aac3-iva* (Additional file 2: Fig. S9).

### ARG-carrying contig analyses

**Fig. 8:**
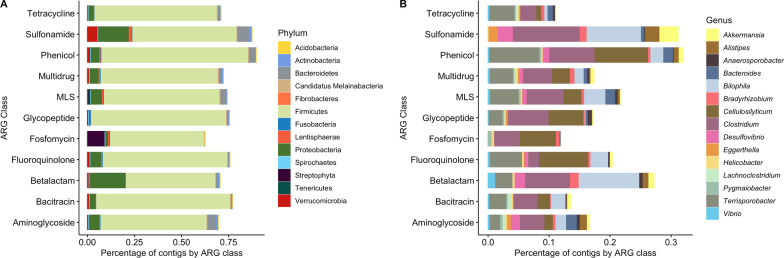
Proportion taxa associated with contigs carrying ARGs (ACCs). **A** Phyla associated with ACCs. **B** Top 15 bacterial genera associated with ACCs

A total of 39,103 contigs carrying ARGs (ACCs) of the most abundant ARG classes (*n* = 11) were analyzed with CAT to predict their bacterial hosts. 73.41% of the contigs were classified at the phylum level. Firmicutes were identified as the most common phylum carrying ARGs (65.16%), followed by Proteobacteria (4.08%), and Bacteroidetes (2.24%) (Fig. 8A). Only 18.67% of the ACCs were taxonomically classified at the genus level. The most common genera carrying ARGs were *Clostridium* (5.22%), *Cellulosilyticum* (3.59%), *Terrisporobacter* (3.24%), and *Bilophila* (1.23%) (Fig. 8B). ACCs of genera of public health interest were identified in a lower proportion of contigs including *Klebsiella* carrying MDR ARGs (0.005%), *Streptococcus* with glycopeptides, MDR and sulfonamide ARGs (0.005%), *Enterococcus* with ARGs to aminoglycosides and beta-lactams (0.005%), *Helicobacter* with resistance to all the antibiotics analyzed but sulfonamides (0.112%), and *Vibrio* with resistance to all classes except for fosfomycin and sulfonamides (0.002%).

In addition, the presence of MGEs and virulence genes was identified in ACCs. Only 3% of the ACCs harbored also MGEs (*n* = 862), while 43 contigs contained virulence genes, and 2 ACCs carried both MGEs and virulence genes. Glycopeptide was the predominant ARG class associated with genes involved in HGT, followed by MDR and MLS (Additional file 2: Fig. S10). The dominant mechanism of HGT associated with ARGs were genes involved in replication/recombination/repair, followed by phages, transposons, and regulation/competence.

A co-occurrence network based on correlations calculated with a matrix of presence/absence of ARGs, MGEs, and virulence genes identified in ACCs was built to observe common contig associations (Additional file 2: Fig. S11). To mention some, the gene *TaeA,* which provides resistance to pleuromutilins, had the highest degree of centrality (*n* = 9) showing connections with *tra* genes involved in plasmid conjugation (*n* = 8) and the intron gene *ltrA*. The gene *vanS* (vancomycin) was correlated with the transposon genes *tnpA* and *tnpB*. Similarly, *aac(3)-II* (aminoglycoside) was associated with the transposon gene *tnpR.*

### Global metagenomic network

**Fig. 9:**
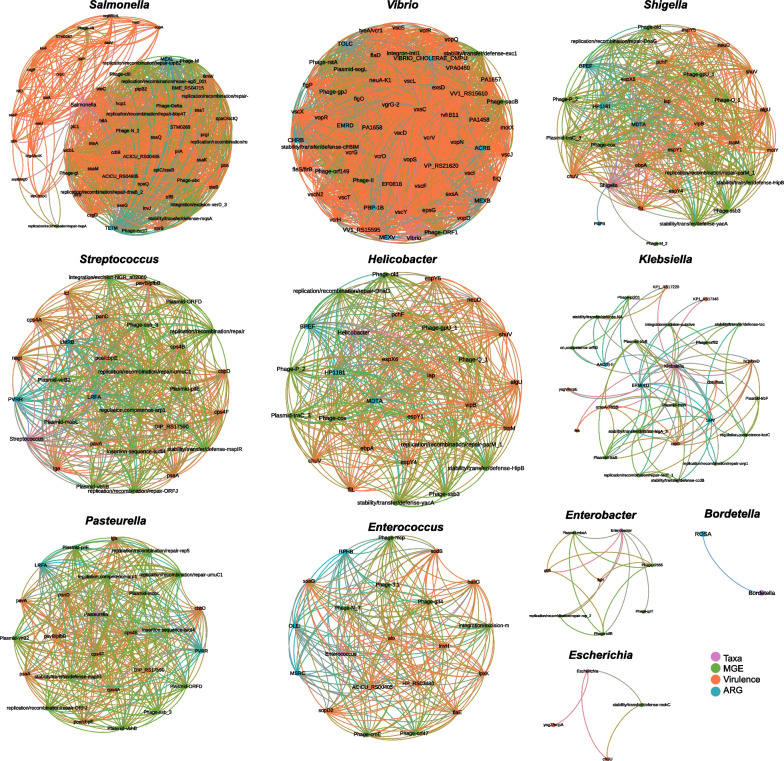
Ego networks of taxa of public and animal health interest identified through mNGS of feces from Galapagos marine iguanas

Correlations between the normalized abundance of microbiome (genera, *n* = 1606), resistome (ARGs, *n* = 244), mobilome (genes, *n* = 1125), and virulome (genes, *n* = 673) were used to depict a global co-occurrence network to identify key associations in the metagenome of the 32 marine iguana samples analyzed through mNGS. The global network had 65 connected components, with an average degree of centrality of 43.3 and modularity of 0.561. In order to identify relevant connections, ego networks from taxa of public and animal health concern were analyzed (Fig. 9; Table 1). Among taxa of public health interest, the most common ARGs belong to the classes MDR and MLS, while phage and effector delivery system genes had the highest number of correlations with these taxa. Notoriously, the genera *Salmonella* and *Vibrio* had the highest degree of centrality, which was dominated by connections with virulence genes, followed by MGEs and ARGs (Fig. 9; Table 1).

**Table 1:** Number of correlations between taxa of public health interest and genes implicated in antibiotic resistance, HGT, and virulence

	*Salmonella*	*Vibrio*	*Shigella*	*Streptococcus*	*Helicobacter*	*Klebsiella*	*Pasteurella*	*Enterococcus*	*Enterobacter*	*Escherichia*	*Bordetella*
ARG	2	8	4	3	3	3	2	3	0	0	1
Aminoglycoside				1		1	1				
Beta-lactam		1				1					
Fluoroquinolone				1			1				
Fosmidomycin											1
MLS		1		1		1		2			
Multidrug	1	5	3		3						
Peptide		1	1								
Rifamycin								1			
Tetracycline	1										
HGT/MGE	17	10	12	13	11	15	12	7	5	1	0
Insertion sequence				1			1				
Integration/excision	1			1		1		1			
Integron		1									
Phage	8	6	7	1	6	2	1	6	3		
Plasmid		1	1	5	1	4	5		1		
Regulation, competence				1		2	1				
Replication/recombination/repair	7		2	3	2	2	3		1		
Stability/transfer/defense	1	2	2	1	2	4	1			1	
Virulence	47	42	15	13	14	8	11	10	2	2	0
Adherence	4	2	1	4	1	1	4			1	
Biofilm			1		1						
Effector delivery system	36	31	6		6	4		3			
Exoenzyme		1		2			1				
Exotoxin	4		1		1			1			
Immune modulation	2			4		2	4	2	1		
Invasion		1	1		1						
Motility		7	2		1			2	1		
Nutritional/Metabolic factor	1		3	2	3	1	2	1		1	
Others				1							
Stress survival								1			
Degree of centrality	66	60	31	29	28	26	25	20	7	3	1

*Salmonella* was correlated with genes encoding the type three secretion system (TTSS) of the *Salmonella* pathogenicity island 1 (SPI-1) (genes, *n* = 13), TTSS (SPI-2) (*n* = 8), and their cognate secreted effectors of TTSS-1 (*n* = 4) and TTSS-2 (*n* = 6). Other delivery systems correlated with *Salmonella* were T6SS (*n* = 1), T4SS secreted effectors (*n* = 1), and those from the *Salmonella* centisome island (SCI) (*n* = 2). Additional virulence genes correlated with the genus *Salmonella* were typhoid toxin (*n* = 3), phospholipase C (*n* = 1), capsule (*n* = 2), hemO cluster (*n* = 1), curli adhesins Agf (*n* = 2), type 1 fimbriae (*n* = 1), and type IV pili (*n* = 1). Only two ARGs were identified in the ego network of *Salmonella*, including the tetracycline gene *tetM* which protects ribosomes and has been priorly associated with transposases, and the MDR gene *MexL* which encodes for an antibiotic efflux pump that confers resistance to disinfecting agents and antiseptics, tetracycline, and macrolides. However, *Salmonella* was part of a bigger cluster in the global network that also contained ARGs conferring resistance to MDR (*emrB*), MLS (*tlrb*), and aminoglycoside:aminocoumarin (*cpxA*) which were indirectly connected to this Enterobacteria.

The genus *Vibrio* also had main connections with genes encoding for a type 3 secretion system (T3SS1; *n* = 20) and its secreted effectors (*n* = 4). Nevertheless, other secretion systems were also correlated with *Vibrio*, including T3SS2 (*n* = 2), virulence-associated secretion (VAS) T6SS (*n* = 1), extracellular protein secretion (Eps) T2SS (*n* = 1), and Rickettsiales *vir* homolog (Rvh) T4SS (*n* = 1). Among other virulence genes connected with *Vibrio* are those encoding for pili (*n* = 2), hyaluronidase (*n* = 1), K1 capsule (*n* = 1), and flagella (*n* = 7). A total of eight ARGs were directly connected with *Vibrio*, including five MDR genes encoding for efflux pumps (*acrB*, *emrD*, *mexB*, *mexV*, and *tolC*), the gene *Vibrio cholerae ompU* that confers resistance to antimicrobial peptides and bile, the gene *chrB* that encode for methyltransferases providing resistance to chalcomycin, mycinamicin, tylosin and lincosamides, and the penicillin-binding protein *PBP-1b* that confers resistance to beta-lactams.

*Shigella, Streptococcus, Helicobacter, Klebsiella, Pasteurella, and Enterococcus* had about half of the connections observed in *Vibrio* and *Salmonella*, while *Enterobacter* and *Escherichia* had a lower number of connections with MGEs and virulence genes and were not linked with ARGs (Table 1). *Bordetella* was correlated only with a fosmidomycin ARG, whereas other bacterial genera of interest did not show connections in the global metagenomic network, including *Staphylococcus*, *Pseudomonas*, *Acinetobacter*, *Aeromonas*, *Campylobacter*, *Neisseria*, *Clostridium*, *Clostridiodes*, *Mycobacterium*, and *Mycoplasma*.

**Fig. 10:**
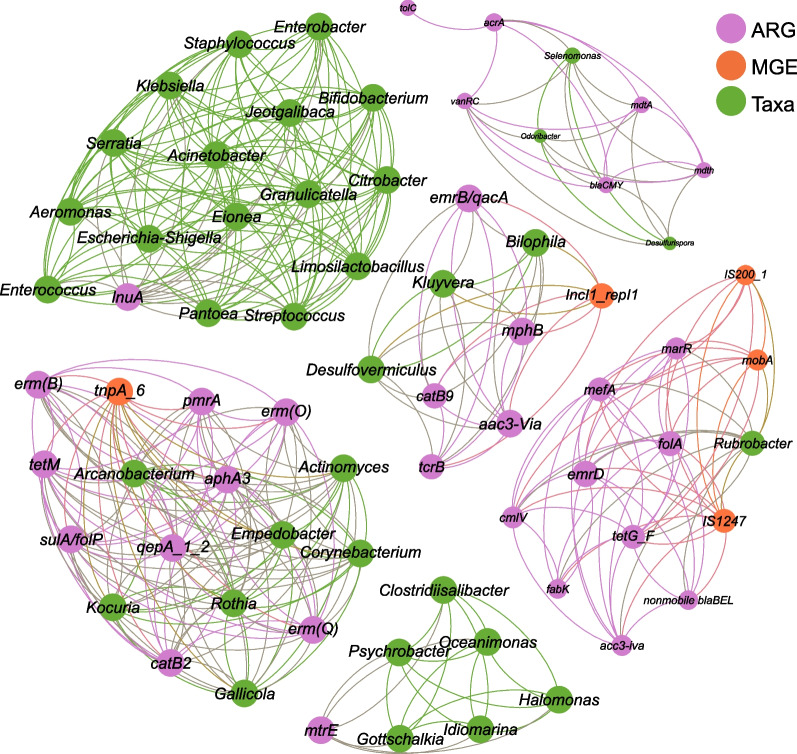
Co-occurrence network between ARGs, MGEs, and bacterial genera identified in the Galapagos marine iguana’s gut. Node size represents the degree of centrality

Correlation networks among bacterial genera, ARGs and MGEs of the 68 samples analyzed with 16S *rRNA* sequencing and SmartChip RT-PCR, resulted in 6 connected components (*modularity* = 0.754, *average clustering coefficient* = 0.97) with an average degree of centrality of 11.72 (Fig. 10). The highest degree of centrality (*n* = 16) was detected in a cluster containing the lincomycin resistant gene *lnuA* and several bacterial taxa including *Escherichia*, *Klebsiella*, *Staphylococcus*, *Enterococcus*, *Aeromonas*, among other genera (Fig. 10). The same number of connections (*n* = 16) was observed in another module containing a transposon gene (*tnpA*), ARGs for aminoglycosides, amphenicol, MLSB, fluoroquinolones, sulfonamide and tetracycline, and commensal taxa (i.e., *Actinomyces*, *Arcanobacterium*, *Corynebacterium*, *Empedobacter*, *Gallicola*, *Kokuria*, and *Rothia*).

### Core metagenome analyses

**Fig. 11:**
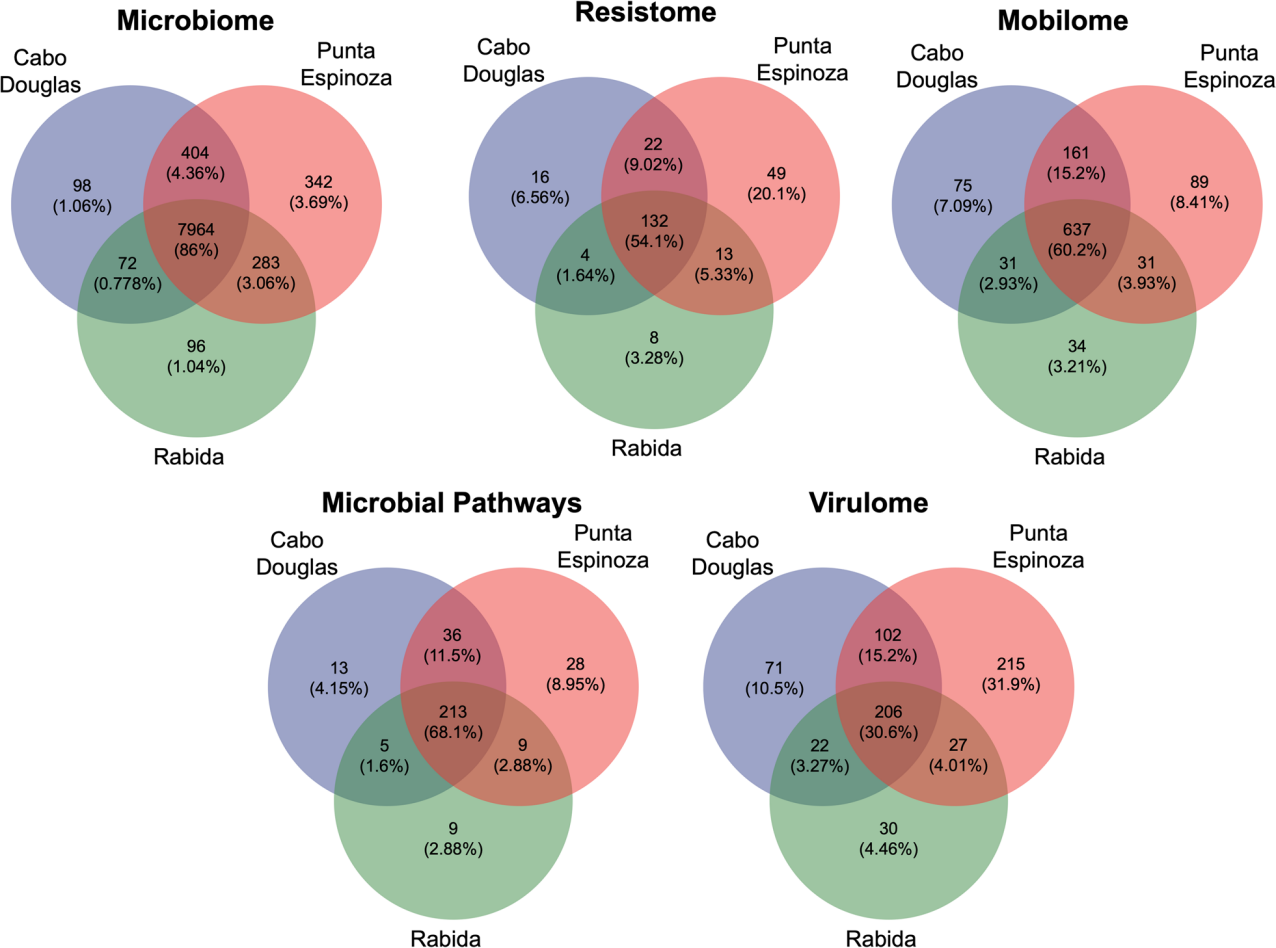
Core metagenome composition of feces from marine iguanas from three colonies

The presence of microbial taxa, genes, and metabolic pathways identified through mNGS were compared between sites. Interestingly, 86% of the taxa were shared between sites showing important similarities in the microbiome composition despite differences in ecological niches (Fig. 11). However, the carriage of accessory genes including those involved in virulence, antibiotic resistance, and HGT revealed a lower percentage of shared features (30.6%, 54.1%, and 60.2%, respectively) (Fig. 11). Importantly, Punta Espinoza showed a greater number of unique features not observed in the other locations, particularly in the resistome and virulome. The functional metagenome also showed that 68% of the metabolic pathways were common among locations, though some metabolic features were unique per location (Rabida 2.88%; Cabo Douglas 4.15%, and Punta Espinoza 8.95%) showing site-specific metabolic adaptations.

## Discussion

Anthropogenic factors such as climatic change, pollution, and antimicrobial usage can affect endangered wildlife populations, particularly in the fragile ecosystem of the Galapagos Islands where marine iguanas are one of the most vulnerable species. A better understanding of the importance of the marine iguana’s microbiota and its natural resistome would enable assessing future impacts of anthropogenic origin to protect this endangered species. Hence, we sought to characterize the marine iguana’s gut microbiome and resistome from two uninhabited islands, Fernandina and Rabida, using culture-independent approaches including 16S *rRNA* sequencing, SmartChip RT-PCR, and mNGS. The most abundant taxa identified in the marine iguana’s gut were Firmicutes (69.4%) which were also the main reservoirs of ARGs (65.16%). Clinically relevant ARGs were identified in the marine iguana’s gut, including those for beta-lactams, colistin, vancomycin, fluoroquinolone, and sulfonamide, though their proportion in their bacterial communities was low (0.006%). Differences in the abundance of 4.53% of the microbial-metabolic pathways were identified among locations showing slight functional microbiome adaptations.

### Niche-specific differences in the marine iguana microbiome

Galapagos marine iguanas from the three colonies had a similar microbiome consisting of 86% genera and 68.1% metabolic pathways shared between sites, showing a convergent microbiome composition with microbial metabolic adaptations. Interestingly, marine iguanas from Cabo Douglas and Rabida had highly similar microbiome and accessory gene composition even though these colonies were distantly located and belonged to two different subspecies. Punta Espinoza, however, exhibited the most diverse metagenomic composition.

Among the three sites, Rabida has the highest visitor carrying capacity with an average of 11.3 groups a day, as compared to Punta Espinoza with 3.6 daily groups and Cabo Douglas which does not allow land visits. Nevertheless, no relationships were identified in the microbiome or resistome based on the number of visitors to these three sites. Lankau et. al., 2012, reported that local exposures influence the iguana’s microbial gut composition in different locations through a process of ecological drift [8]. Differences associated with the diet were evidenced by the differential abundance of microbial metabolic pathways in the marine iguana gut between locations. For instance, iguanas from Fernandina have a higher abundance of pathways associated with the use of glucose and triacylglycerol, synthesis of heptose sugars like the ones found in cell surface polymers of bacteria including lipopolysaccharides or the O-antigen, and vitamin B6 biosynthesis; while lizards from Rabida had higher use of hexitol, a sugar found naturally in some plants, and degradation of amino acids for the formation of ATP.

Fernandina is surrounded by the cold Cromwell current which allows a more diverse marine ecosystem with higher availability of seagrass than the ones found in Rabida where warmer currents converge. Sea surface temperatures are negatively correlated with the length of algae pastures and the size of marine iguanas [5]. Iguanas from Fernandina Island are large-sized while the ones from Rabida are considered medium-sized subspecies [17]. In our study, SVL measurements of 45 specimens (Cabo Douglas, *n* = 20; Punta Espinoza, *n* = 20; Rabida, *n* = 5) corroborated differences in body size between locations (Cabo Douglas, *SVL* = 317.9 ± 65.8; Punta Espinoza, *SVL* = 354 ± 46.9; Rabida, *SVL* = 214 ± 23.02). Moreover, temperature increases have been associated with changes in the gut microbiome characterized by a decrease of Firmicutes and an increase of predicted pathogenic clades in wild-caught western fence lizards (*Sceloporus occidentalis*) [52].

Despite these factors, the high diversity of microbiome and resistome observed in Punta Espinoza is intriguing and further analyses are needed to explain these differences, including algae composition, sea temperature, and metabolome analyses of feces to identify compounds that can influence the gut microbiome functional profile.

### Marine iguana microbiome composition

The average marine iguana’s gut microbiome was dominated by Firmicutes, followed by Proteobacteria and Bacteroidetes. Similar composition has been observed in other lizards [52, 53] and iguanas [6, 7]. Studies carried out in Galapagos marine iguanas found similar proportion of Firmicutes (60–75.1%); however, different proportions of Proteobacteria (< 3.1–8.9%) and Bacteroidetes (8.2–22.5%) were reported, respectively [6, 7]. These differences could be attributed to several factors including, different sampling sites (San Cristobal, Santa Fe, Plaza Sur, and Fernandina), date of collection (August–September 2009 [cold-dry season]; and January 2001 [La Niña]), and sequencing technology (454 pyrosequencing) [6, 7].

In this study we identified important differences in the proportion of bacterial taxa between 16S *rRNA* sequencing and mNGS, as priorly reported in an investigation that used chicken gut samples as a model [54]. That study showed that 16S *rRNA* sequencing only detects part of the gut microbiota than the one observed with shotgun sequencing and that more biologically important taxa were identified through mNGS [54]. Moreover, current databases limit the identification of microbial species found in marine iguanas since the wildlife microbiome composition is less studied than the one of humans and domestic animals. Nevertheless, Kraken2 was able to classify most of the taxa at the kingdom and phylum levels in the metagenomes (mNGS). Future comparative studies should account for differences in location, season, and sequencing approaches to accurately identify changes in the marine iguana’s gut microbiome composition.

The functional microbiome profile unravels the symbiotic relationship of the gut microbiome with the macrophytic algae-consuming marine iguanas. As observed in other herbivorous species, the gut microbiome of marine iguanas is essential for the biosynthesis of proteins, energy, and vitamins. A prior study identified several carbohydrate degrading enzymes, genes associated with sulfur metabolism and dehalogenation enriched or unique to the marine iguanas as compared to other herbivorous hosts [7]. Though, our study is the first one that integrates metabolic pathways to unravel the functionality of the gut microbiome of the Galapagos marine iguanas.

### Marine iguana resistome composition

Notably, the resistome composition of the Galapagos marine iguanas from uninhabited islands revealed a low abundance of ARGs. Similar results have been observed in wildlife from isolated areas [55] including Galapagos land iguanas from Santa Fe Island [12], Antarctic penguins [56], and marine-feeding gulls from remote Alaska [57] where no or limited carriage of antibiotic-resistant bacteria have been identified. A recent comparison of the abundance of 21 ARGs of the seven drug classes in Galapagos-giant tortoise’s fecal DNA revealed significant differences in the abundance of ARGs present in animals from Santa Cruz (the most populated island in the archipelago) as compared to animals from a remote area of the Isabela island [58]; that study highlights the importance of human activities in the levels of resistance as well as the role of giant tortoises as ARG spreaders into the environment. Wheeler et. al., 2012, analyzed the resistance of Enterobacteriaceae isolates from land iguanas, marine iguanas, giant tortoises, and seawater, outlining that resistant strains were only identified in seawater close to a public use beach and from animals from touristic sites but not from those found in isolated islands [13]. As a result, wildlife could be considered sentinels of antibiotic-resistant bacteria in different habitats. Since the Galapagos have human populations in four islands occupying 3% of the landmass, this setting provides an ideal model that would enable identifying impacts of anthropogenic nature, including antibiotic usage in humans and domestic animals. Future studies should keep monitoring the resistome in the Galapagos Islands using One Health strategies which include the human, environmental, and animal sources.

The most common ARGs identified in the marine iguanas were those conferring resistance to aminoglycosides, MDR, vancomycin, and tetracycline through SmartChip RT-PCR. Meanwhile, the analysis of assembled contigs from mNGS showed that the most abundant ARG classes present in the marine iguana’s resistome were glycopeptide (vancomycin), MDR, tetracycline, and bacitracin, though ARGs conferring resistance to 22 drug classes were identified in the marine iguana’s resistome. Differences between these two approaches were expected as SmartChip RT-PCR explores the targeted presence of a group of genes; meanwhile, mNGS is an untargeted methodology used to explore more broadly the resistome composition. In addition, DeepARG, used to identify ARGs in assembled metagenomes, applies machine learning to improve the prediction of ARGs resulting in higher precision and recall of the resistome composition.

Priorly, resistance to ampicillin, tetracycline, and trimethoprim-sulfamethoxazole was identified in Enterobacteria isolated from marine iguanas from Plaza Sur which is located close to human populations, while isolates from Santa Fe, an uninhabited island, where susceptible to the 12 antibiotics tested [13]. Furthermore, non-dominant Enterobacteria isolated from land iguanas showed resistance to ampicillin, tetracycline, nalidixic acid, and gentamicin [12]. A recent study focused on the characterization of wildlife, human and environmental resistome in the Galapagos using mNGS found that marine iguanas (*n* = 8) exhibited low levels of ARGs (2.98E-03 ± 2.18E-03), where the top 3 ARGs were those from the classes beta-lactam (*cepA*), aminoglycoside (*cepA-49*) and lincosamide (*cepA-29*) [16]. Here, twice the amount of ARGs of that previous study was detected in the marine iguana’s metagenome, though differences in resistome databases and bioinformatic approaches explain these disparities [16]. A lower abundance of MGEs, however, was identified in this study compared to Grube et. al, 2021 report (0.03% vs. 0.06%), where integrons, plasmids, insertion sequences, and transposases were identified. ARGs associated with microbial resistant threats including ESBLs and vancomycin resistance were identified in the marine iguana’s gut. Beta-lactam resistant genes were also identified priorly in the marine iguana’s resistome including class A, *OXA-9*, *PBP1B*, *penA*, and *ccrA* [16].

### Bacterial hosts of ARGs

Network and ACC analyses, however, revealed that most bacterial hosts of these ARGs were present in commensal bacteria. Such is the case of *Rubrobacter*, a halotolerant genus, which showed the highest number of connections with ARGs to multiple drugs and MGEs in the co-occurrence network. Whole-genome sequencing of *Rubrobacter radiotolerance* revealed that this taxa harbor several virulence factors, ARGs, MGEs, and genes involved in the biosynthesis of antibiotics, showing the potential of environmental bacteria to resist and produce antibiotics [59]. Opportunistic and true pathogens were also associated with ARGs including *Salmonella*, *Escherichia*, *Klebsiella*, *Streptococcus*, *Enterococcus*, *Helicobacter,* and *Vibrio*.

Interestingly, taxa of public and animal health interest identified in the marine iguana’s gut metagenome showed complex associations with virulence genes, MGEs, and ARGs. Despite the distance of marine iguanas from Rabida and Fernandina from human populations, potential pathogens were associated with several ARGs encoding efflux pumps, target protection, inactivating enzymes, and porins conferring resistance to multiple drugs including aminoglycosides, beta-lactams, fluoroquinolones, fosfomidomycin, MLS, peptides, tetracycline, and rifamycin. *Salmonella* and *Vibrio* showed the most complex networks with a predominance of connections with genes encoding for effector delivery systems and bacteriophages, indicating their potential pathogenicity and genomic plasticity. Reptiles are common reservoirs of *Salmonella* and have been associated with risk of enteric disease in humans [60]. Drug-resistant *Salmonella* serotype Typhi is considered a serious threat by the CDC in the USA, where increased resistance to ciprofloxacin (74% of the isolates) and the identification of strains resistant to all but two drug classes (macrolides and carbapenems) have been observed in the USA and Pakistan [61]. Here, *Salmonella* from marine iguanas was correlated with resistance to tetracycline, macrolides, disinfecting agents, and antiseptics, raising concerns about the spread of AR in wildlife from the Galapagos. However, phenotypic analyses of *Salmonella enterica* from marine iguanas of a public beach on San Cristobal Island showed the susceptibility of these bacteria to 12 antibiotics [14]. The identification of ARGs in the genome is not always a predictor of phenotypic resistance and further confirmation is needed. *Vibrio* was correlated with multiple MGEs, including phages, integrons, and plasmids which were also connected to ARGs conferring resistance to beta-lactams, MLS, peptide, and MDR. MGEs play an important role in the acquisition of ARGs in *Vibrio* contributing to the emergence of MDR and extended antimicrobial resistance (XDR) of this genus, which has been extensively reviewed [62]. Through ACC analyses, *Vibrio* was identified in contigs carrying resistance to 9 classes of antibiotics (i.e., aminoglycoside, bacitracin, beta-lactam, fluoroquinolone, glycopeptide, MLS, MDR, and tetracycline) revealing that more ARGs are present in this bacterial species.

Other Enterobacteria identified in the iguana’s gut metagenome carrying ARGs were *Klebsiella* and *Shigella*. Drug-resistant *Klebsiella*, particularly carbapenem-resistant, represents a serious threat to public health associated with high mortality in humans [61]. *Klebsiella* from marine iguanas was correlated with ARGs to aminoglycoside (*aac(3)-IIa*), beta-lactam (*SHV*), and MLS (*erm-41*). The gene *aac(3)-IIa* found in *Klebsiella* is plasmid-encoded and inactivates aminoglycosides such as gentamycin, kanamycin, tobramycin, neomycin, and apramycin. *SHV* is also plasmid-associated and encodes for beta-lactamases with variants that range from narrow to extended-spectrum [63], though allelic variants of this gene were not identified in this study. The identification of *Shigella* in marine iguanas should be confirmed through bacterial isolation and sequencing since this genus has not been associated with reptile reservoirs priorly. However, three MDR ARGs (*mdtA*, *bpef*, and *hp1181*) and a peptide gene (*pgpb*) were correlated with the genus *Shigella*. The gene *mdtA* is part of a complex that confers resistance against novobiocin and deoxycholate, *bpef* encodes for an efflux pump that provides resistance to chloramphenicol and trimethoprim, while *hp1181* is a translocase involved in the efflux of tetracycline, nitroimidazole, and fluoroquinolones. The gene *pgpb* found in *Shigella* encodes for a lipid A phosphatase that protects the cell from peptides. The use of multiple approaches to identify associations between bacterial taxa, MGEs, and ARGs is critical to understanding the dynamics of antibiotic resistance in the microbiome of wildlife. Future studies should focus on identifying changes in the associations between microbial taxa and genes in function of the weather and distance to human populations in order to comprehend the impact of these factors on bacterial resistance and pathogen carriage.

### Study imitations

A drawback of the present study relies on convenience sampling and the amount of fecal material available for DNA extraction and further metagenomic characterization. DNA concentrations were relatively low, which limited the detection of low abundant taxa and ARGs. Despite this limitation, the high number of samples analyzed (*n* = 68) makes this study one of the most comprehensive ones on the microbiome and resistome of the Galapagos marine iguanas. Moreover, convenience sampling was carried out in uninhabited islands providing valuable information on the natural resistome and microbiome of marine iguanas; nonetheless, future studies should include animals living in close proximity to human settings to evaluate the anthropogenic influence on the abundance of ARGs and MGEs, as well as changes in the microbiota composition. Finally, better identification of bacterial hosts of ARGs could be possible through enrichment and long-read sequencing as proven priorly [64].

## Conclusion

The Galapagos marine iguanas from remote islands harbor a diverse pool of ARGs, although a low abundance of these genes was observed in their gut metagenome. Niche-specific adaptations were evidenced in microbial accessory genes (i.e., ARGs, MGEs, and virulence) and metabolic pathways, though a highly preserved microbiome composition was identified among locations. ARGs were mostly associated with commensal bacteria. However, the presence of AR pathogens, such as *Salmonella* and *Vibrio* in marine iguanas raises concerns about the dispersion of microbial resistant threats in pristine areas, highlighting wildlife as sentinel species to identify the impact of AU.

## Supplementary Information

**Additional file 1.** (Excel format). Normalized abundance of ARGs and MGEs identified with SmartChip RT-QPCR

**Additional file 2.** (PDF format). Supplementary figures S1–S11

**Additional file 3.** (Excel format). Differentially abundant taxa classified with Kraken2 (mNGS) and analyzed with ANCOM-BC

**Additional file 4.** (Excel format). Microbial metabolic pathways relative abundance (based on reads per kilobase) and classification

**Additional file 5.** (csv format). Normalized abundance of genes conforming resistome, mobilome and virulome identified in fecal metagenomes from marine iguanas

## Data Availability

The datasets and analyses supporting the conclusions of this article are available in the GitHub repository, [Galapagos marine iguana’s gut metagenome in https://github.com/karla-vasco/metagenome_marine-iguana].

## Abbreviations

ACC: Antibiotic-resistant gene carrying contig
ANCOM-BC: Analysis of compositions of microbiomes with bias correction
AR: Antibiotic resistance
ARG: Antibiotic resistance gene
AU: Antibiotic use
CAT: Contig annotation tool
CT: Cycle threshold
DNA: Deoxyribonucleic acid
GC: Genomic copy
HGT: Horizontal gene transfer
MDR: Multidrug
MaAsLin2: Microbiome multivariable associations with linear models
MGE: Mobile-genetic element
MLS: Macrolide-lincosamide-streptogramin
mNGS: Metagenomic next-generation sequencing
ORF: Open-reading frame
OTU: Operational-taxonomic unit
PCoA: Principal coordinate analysis
PERMANOVA: Permutational multivariate analysis of variance
PERMDISP: Permutational analysis of multivariate dispersion
RA: Relative abundance
RT-PCR: Real-time polymerase chain reaction
VFDB: Virulence factor database
