# Supplementary material for: Characterization of the gut microbiome and resistome of Galapagos marine iguanas (Amblyrhynchus cristatus) from uninhabited islands
Source: Anim Microbiome. 2022 Dec 14;4:65. doi: 10.1186/s42523-022-00218-4 (PMC9749353; doi:10.1186/s42523-022-00218-4)
Supplement: Supplementary file 2 — Additional file 2. (PDF format). Supplementary figures S1–S11 [file 42523_2022_218_MOESM2_ESM.pdf]

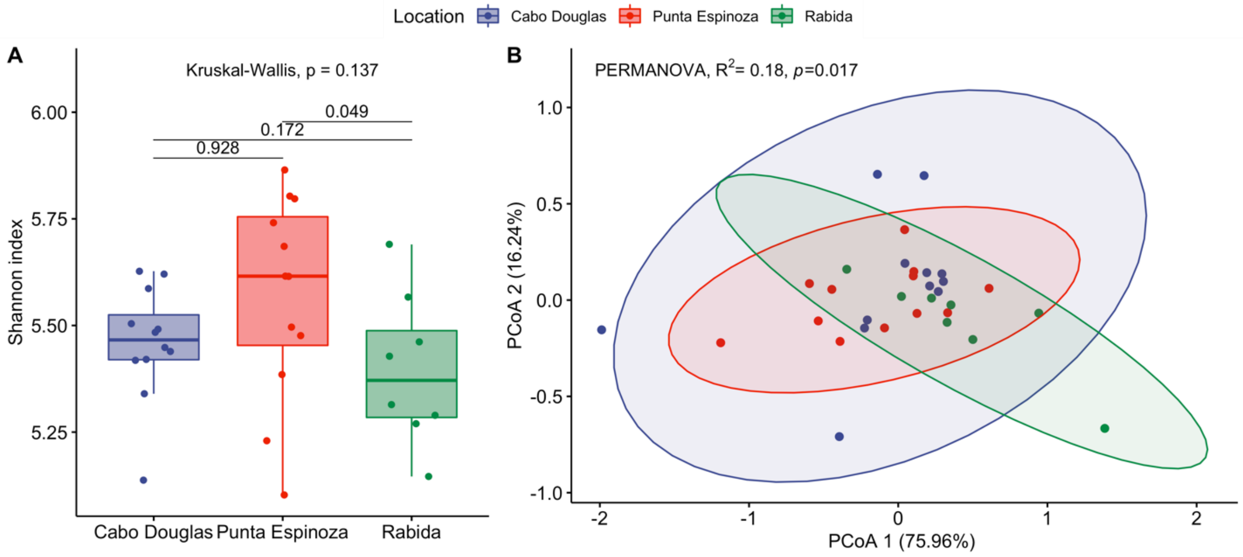

Figure S1. Diversity of marine iguana's gut metagenomes analyzed with Kraken2. A) Shannon Index. B) PCoA of the Bray-Curtis dissimilarity.

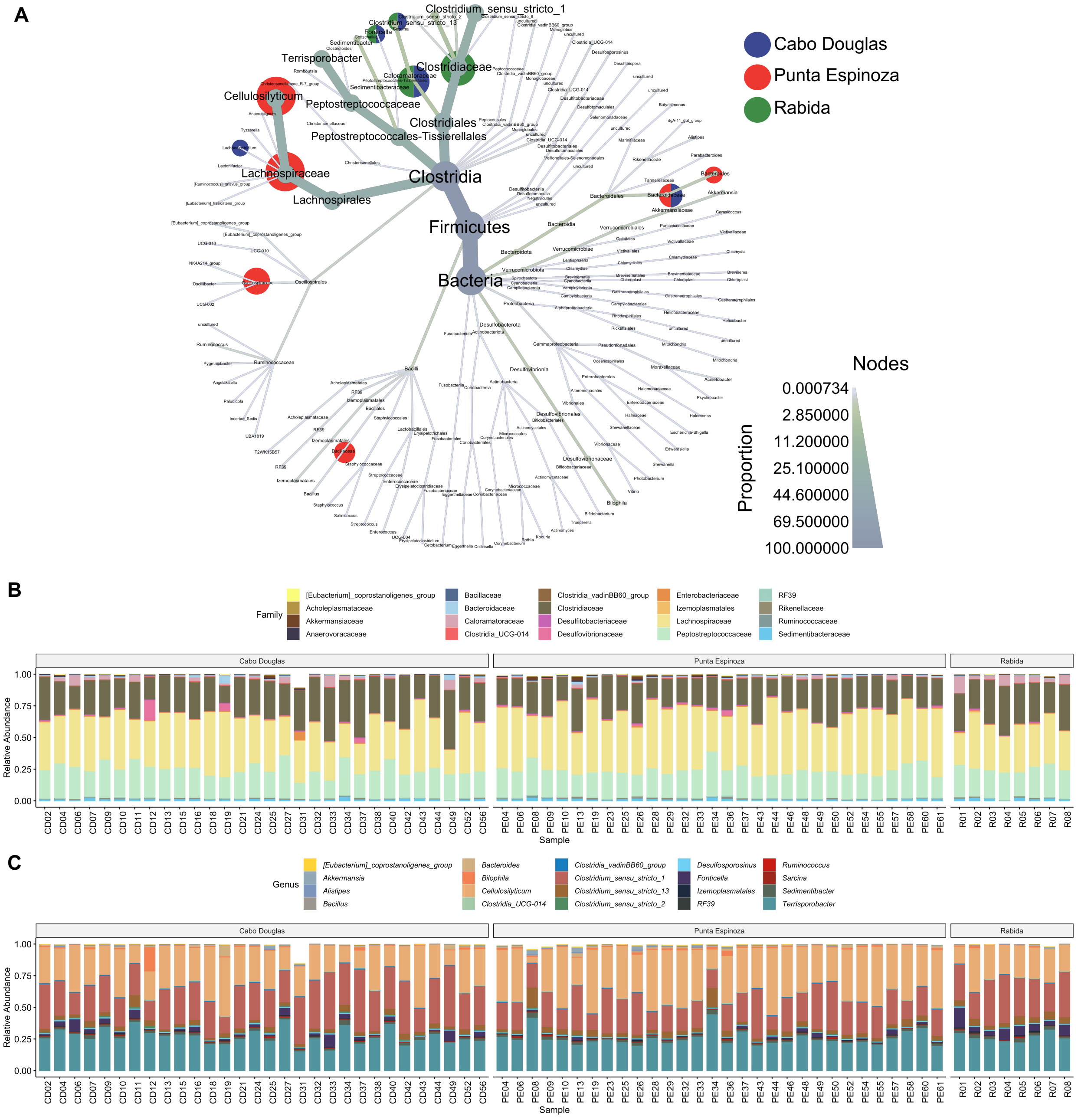

Figure S2. Gut microbiome composition (*16S rRNA* sequencing) of marine iguanas from Fernandina and Rabida Islands. A) Heat-tree showing the proportion of taxa from phylum to genus, taxa significantly higher is highlighted for each location/s. B) Relative abundance of the top 20 most abundant families per sample. C) Relative abundance of the top 20 bacterial genera.

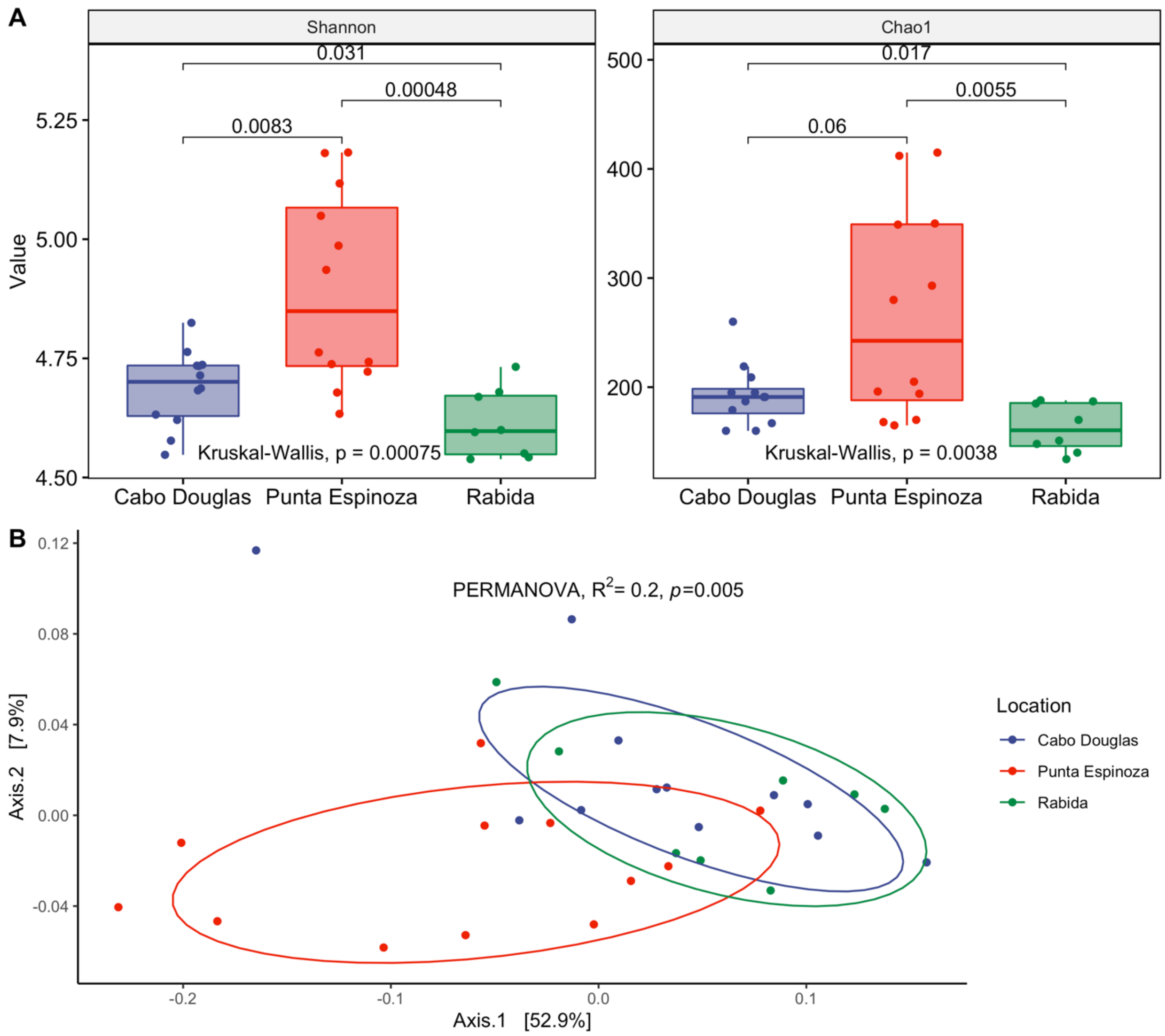

Figure S3. Microbial-associated metabolic pathway's diversity. A) Alpha diversity calculated with the indices Shannon and Chao1. B) Beta-diversity analyzed in a PCoA of a Bray-Curtis dissimilarity matrix.

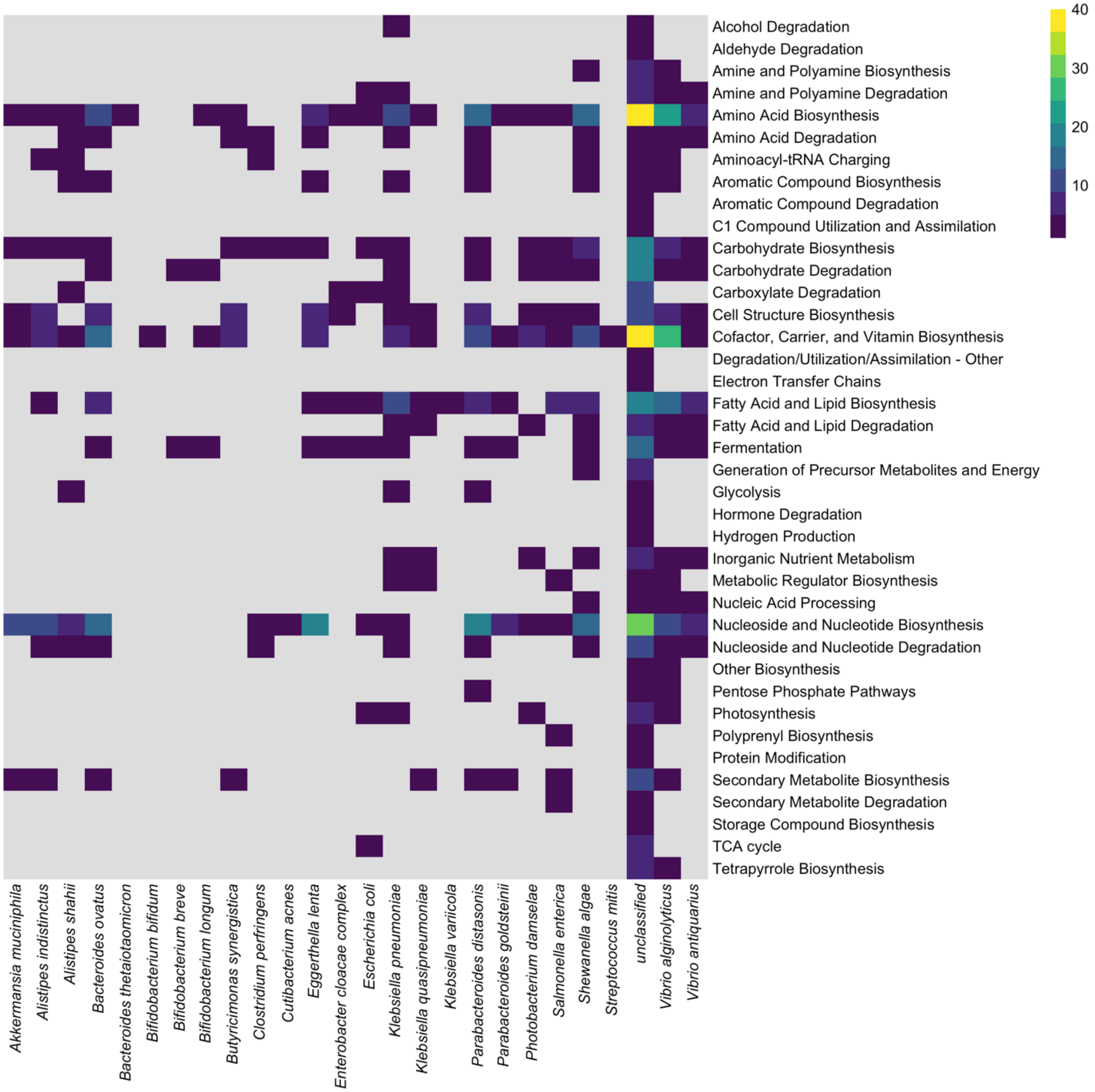

Figure S4. Heatmap showing the number of microbial-metabolic pathways per bacterial species.

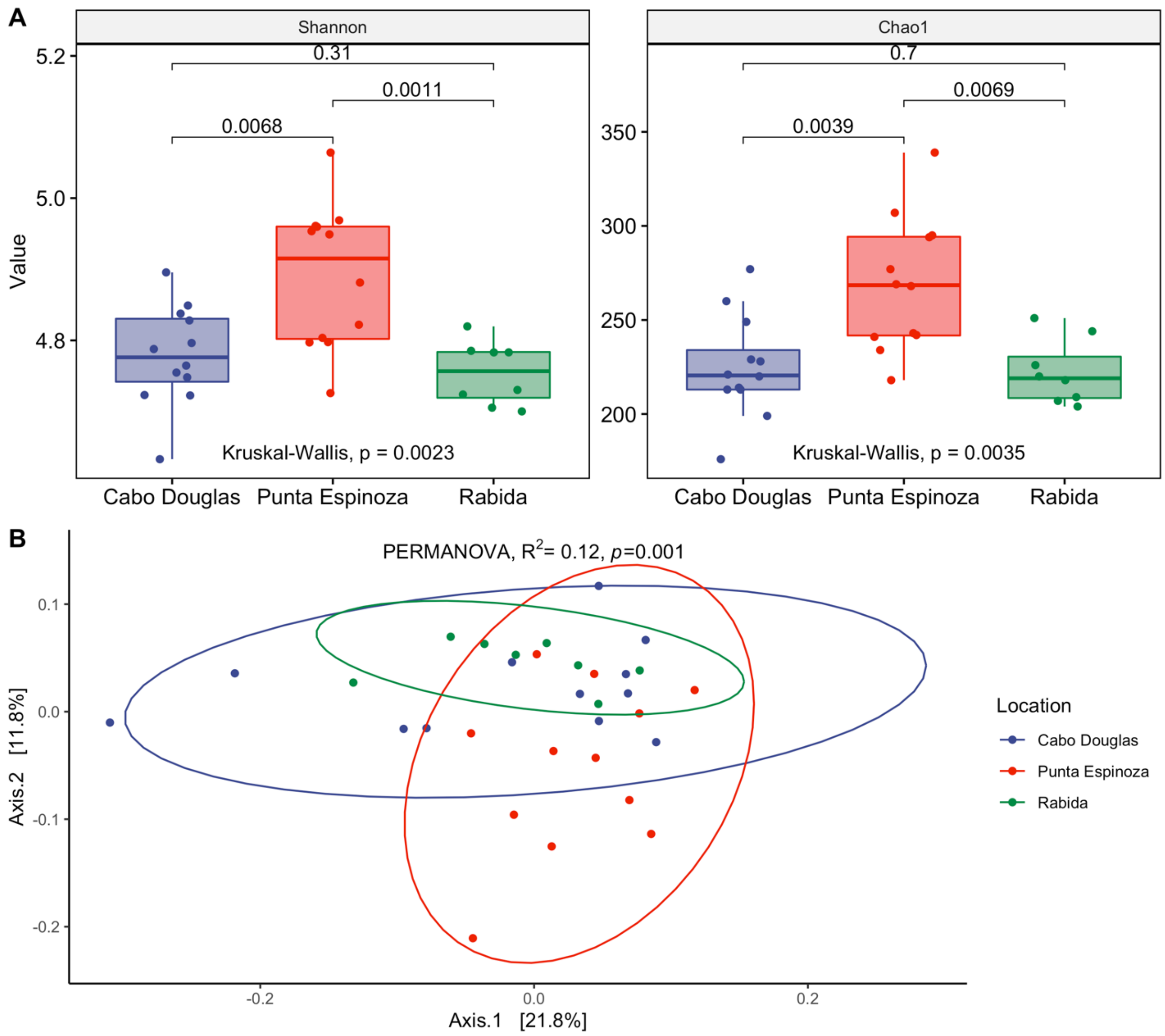

Figure S5. Resistome alpha and beta diversity. A) Alpha diversity calculated with the indices Shannon and Chao1. B) Beta-diversity analyzed in a PCoA of a Bray-Curtis dissimilarity matrix.

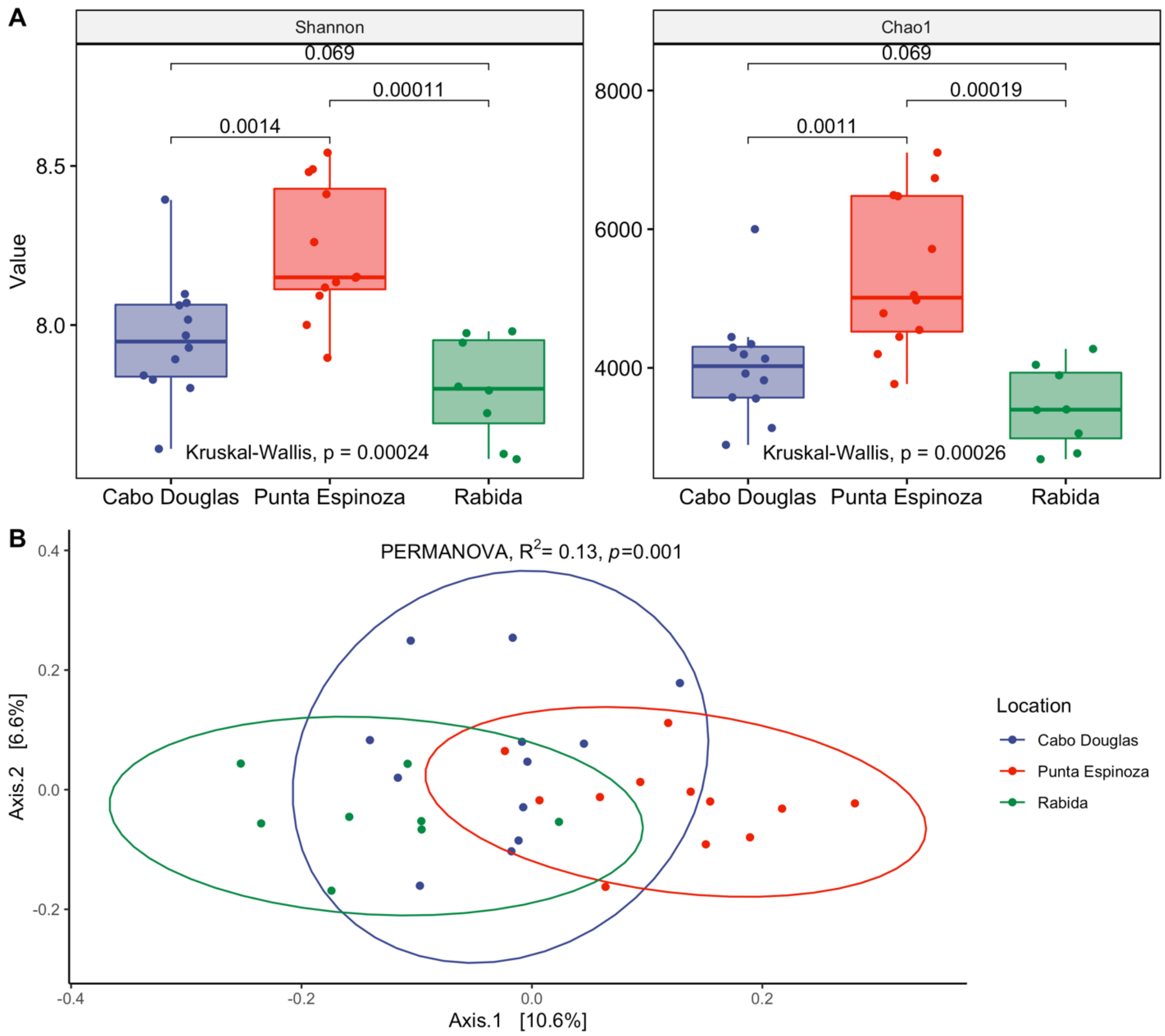

Figure S6. Mobilome alpha and beta diversity. A) Alpha diversity calculated with the indices Shannon and Chao1. B) Beta-diversity analyzed in a PCoA of a Bray-Curtis dissimilarity matrix.

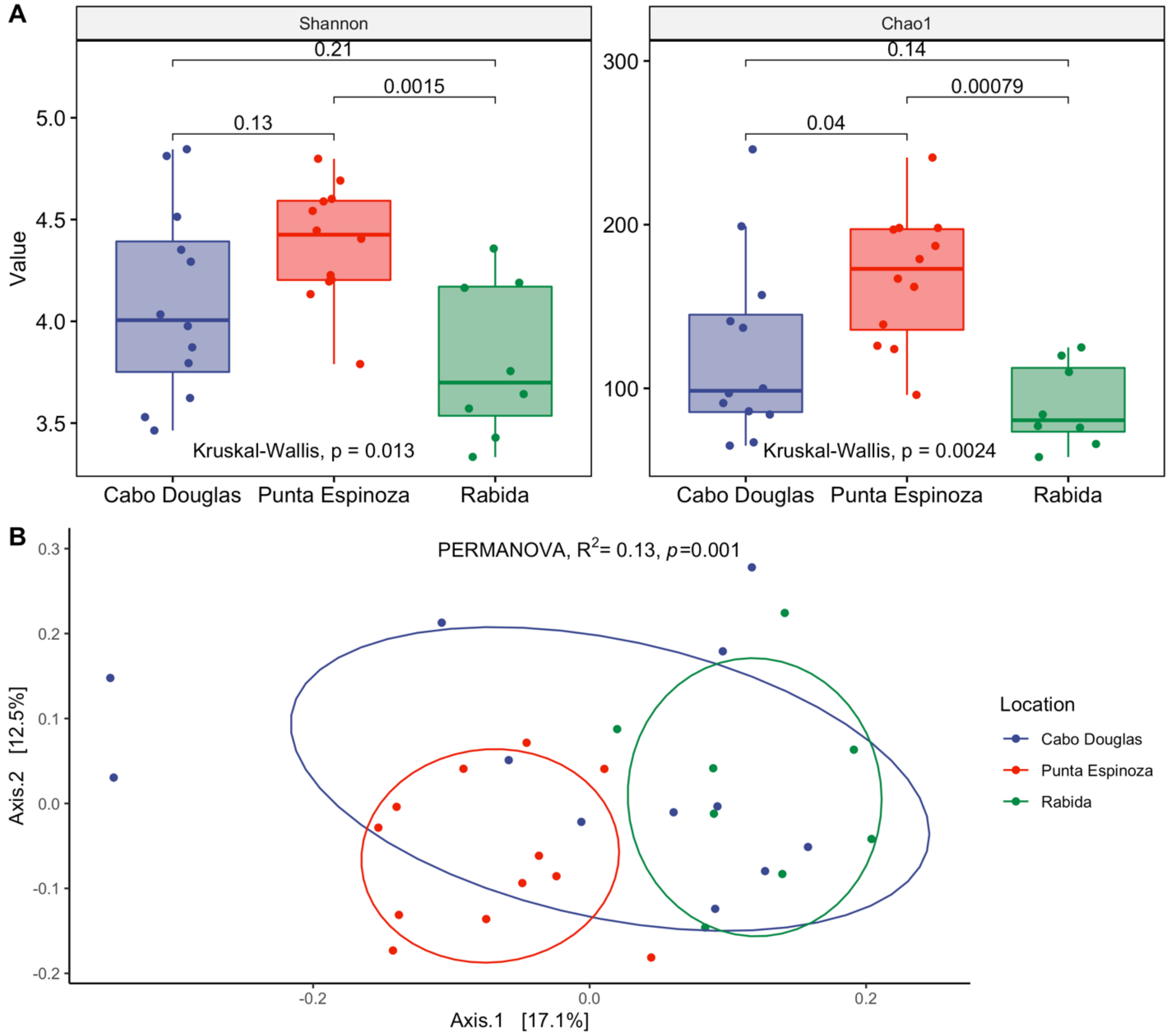

Figure S7. Virulome alpha and beta diversity. A) Alpha diversity calculated with the indices Shannon and Chao1. B) Beta-diversity analyzed in a PCoA of a Bray-Curtis dissimilarity matrix.

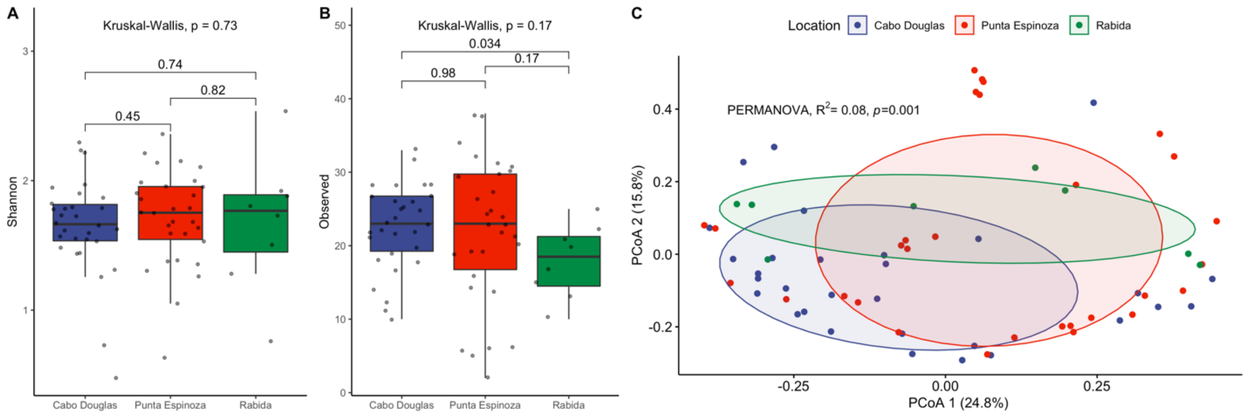

Figure S8. Alpha and beta diversity of the resistome/mobilome detected with SmartChip RT-PCR. (A) Shannon index; (B) Observed number of genes; and (C) Bray-Curtis dissimilarity PCoA.

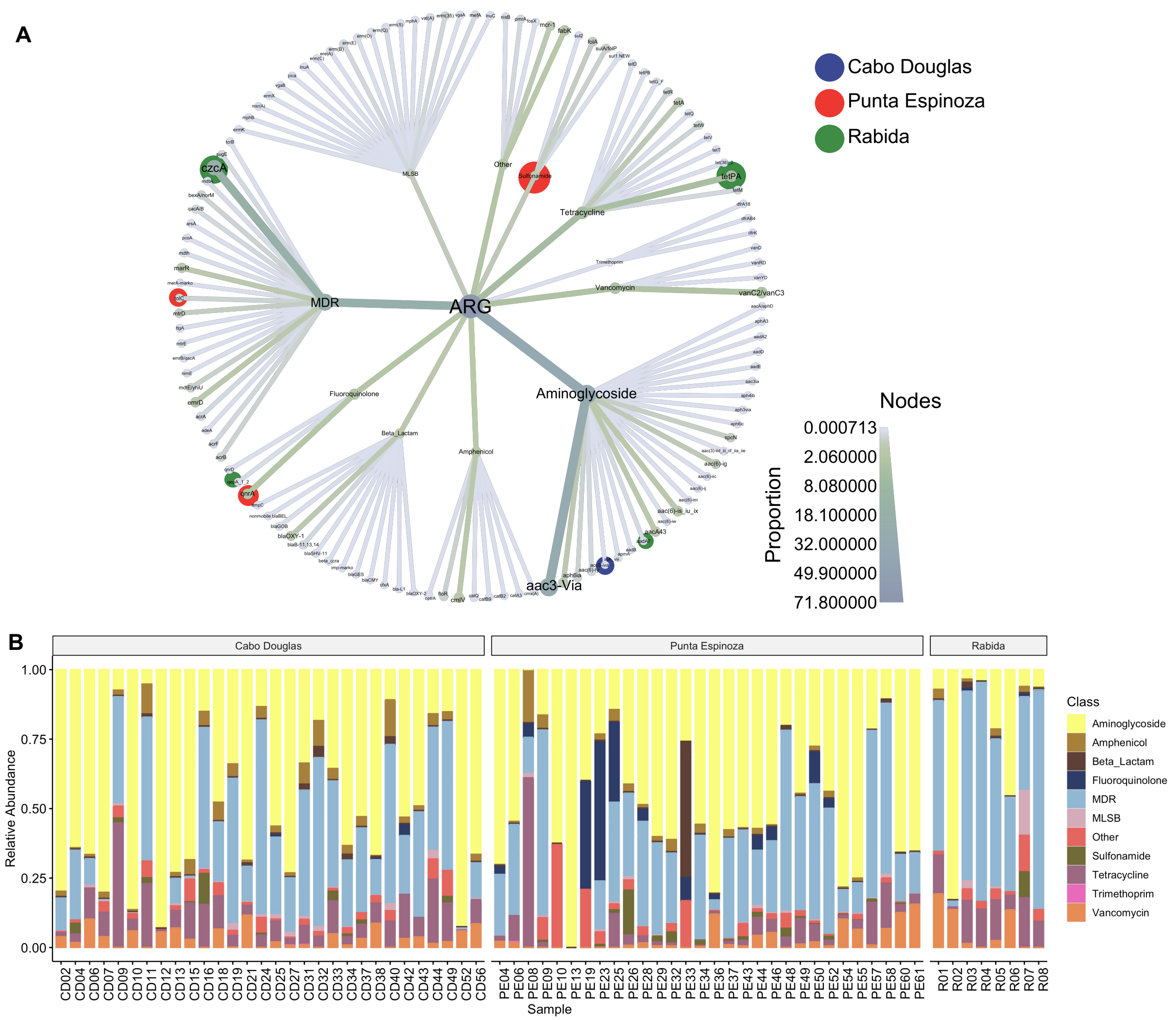

Figure S9. Gut resistome composition identified with SmartChip RT-PCR. A) Heat-tree showing the proportion of ARGs and differentially abundant ARGs among locations. B) Relative abundance of ARGs classes per sample and clustered by location.

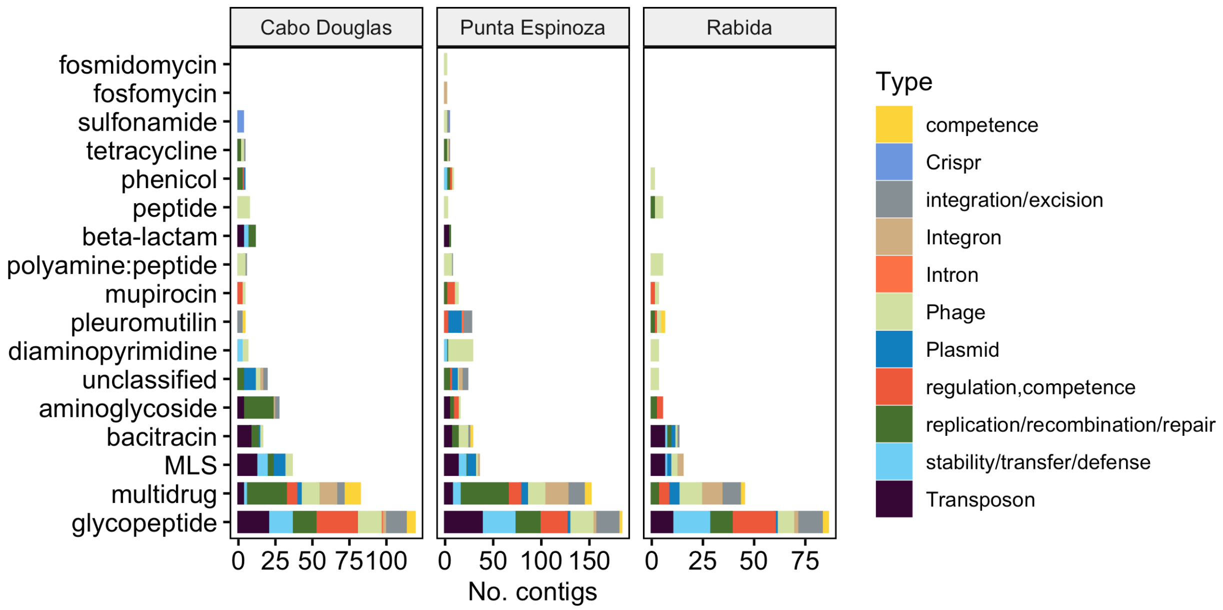

Figure S10. Number or ACC harboring genes involved in HGT by ARG class and location.

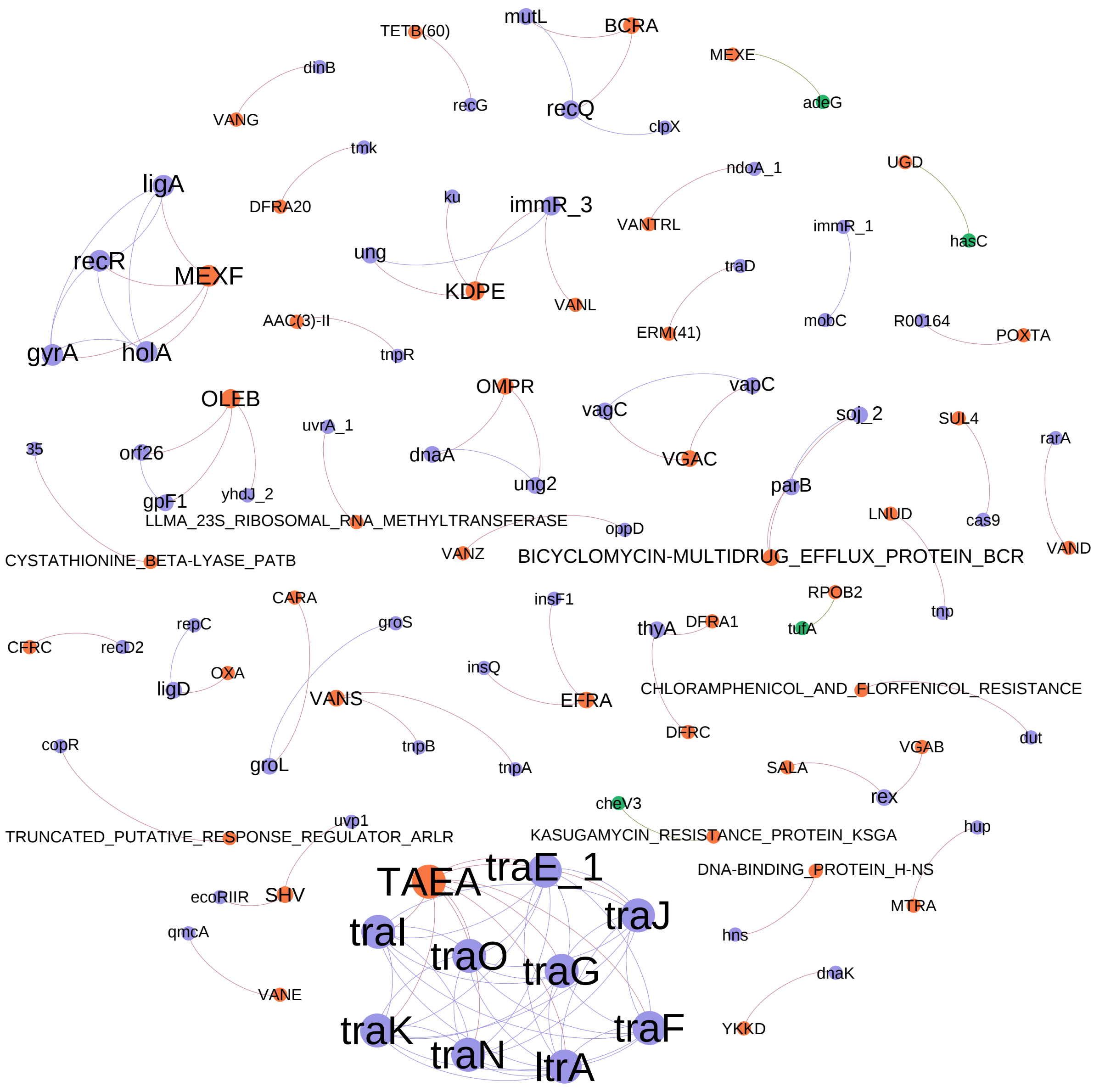

Figure S11. Co-occurrence network of ACCs between ARG, MGE, and virulence genes. Edges represent a correlation ≥ to 0.5 and a p-value < 0.01. Correlations were calculated based on a presence/absence matrix per contig. ARG = orange, MGE = purple, Virulence = Green.
